# The neutrophil-to-lymphocyte ratio in rheumatoid arthritis: The dual perspectives from literature and clinic

**DOI:** 10.1097/MD.0000000000044554

**Published:** 2025-09-19

**Authors:** Yang Li, Jian Liu, Yuedi Hu, Chengzhi Cong, Yiming Chen, Yanyan Fang

**Affiliations:** a Department of Rheumatology, The First Affiliated Hospital of Anhui University of Chinese Medicine, Hefei, China; b First Clinical Medical School, Anhui University of Chinese Medicine, Hefei, China; c Institute of Rheumatology, Anhui Academy of Chinese Medicine, Hefei, China.

**Keywords:** biomarker, clinical investigation, literature, neutrophil-to-lymphocyte ratio, rheumatoid arthritis

## Abstract

The aim of this study was to elucidate the research direction and application value of neutrophil-to-lymphocyte ratio (NLR) as an emerging inflammatory marker in rheumatoid arthritis (RA) from both literature and clinical perspectives. Firstly, we made an extensive analysis in terms of the number of publications, highly cited literature, co-cited references, and keywords. Subsequently, a retrospective exploration of clinical data of 1490 patients with rheumatic diseases admitted to the First Affiliated Hospital of Anhui University of Chinese Medicine was conducted. Logistic regression models were used to explore the independent predictive role of NLR in different scenarios. Nomogram was developed and the model was clinically evaluated by recipient operating characteristic (ROC) curves, calibration plots and decision curve analysis. In addition, association rule analysis and Mantel test were used to determine associations between NLR and clinical characteristics and self-perception of patients. A total of 544 articles were retrieved, focusing on the pathophysiology and clinical studies of RA-NLR, such as “disease activity,” “inflammation” and “classification.” Retrospective analysis demonstrated that NLR, erythrocyte sedimentation rate (ESR) and C-reactive protein (CRP) levels were significantly higher in RA patients than in non-RA patients. Logistic regression models identified gender, age, NLR, ESR and CRP as independent predictors of RA. The ROC curve determined a cutoff value of 2.258 for NLR and a maximum area under the curve of 0.736. Both association rule analysis and Mantel test showed that NLR was highly correlated with inflammatory markers such as ESR, CRP, and self-perception scale scores before and after treatment. NLR > 2.258 was a meaningful risk factor for moderate-to-severe pain and higher disease activity, which predictive reliability was further confirmed in subgroup stratification analyses. NLR, as a novel inflammatory marker, correlates with clinical characteristics and self-perception of RA patients, acting as an independent predictor of RA diagnosis and activity assessment.

## 1. Introduction

Rheumatoid arthritis (RA) is a common chronic systemic autoimmune disease, with clinical manifestations such as morning stiffness, swelling, and pain in joints, and in severe cases, bone erosion, joint deformity, and loss of function can occur.^[1]^ It is reported that the global prevalence of RA is about 1%, and about 0.32% to 0.36% in China, which is increasing year by year, and the disability rate is also high, which seriously affects the quality of life of patients.^[2]^ Because the disease state of RA patients is characterized by high activity, progression and recurrence, clinicians often face great difficulties in choosing therapeutic drugs. We emphasize the concept of early, rapid, and accurate diagnosis,^[3]^ which will allow the identification of patients at high risk of RA progression and assist in the early implementation of standardized interventions. Therefore, it is crucial to find more novel and feasible biomarkers capable of accurately identifying early RA risk and determining disease status.

Currently, the clinical diagnosis and treatment of RA are still centered on immune inflammation. Two important serum markers for diagnosis, rheumatoid factor (RF) and anti-citrullinated protein antibodies (anti-CCP), are often negative in early RA, with a lack of sensitivity and specificity.^[4]^ Erythrocyte sedimentation rate (ESR) and C-reactive protein (CRP), 2 nonspecific classical indicators of inflammation, are susceptible to a variety of factors such as infection and acute stress.^[5,6]^ In the pathogenesis and progression of RA, neutrophils and lymphocytes play an important role in innate and adaptive immunity.^[7,8]^ The hyperinflammatory state in patients with active RA promotes the progression of RA by stimulating anti-apoptotic cytokines and granulocyte colony-stimulating factors thereby enhancing neutrophil activation and proliferation, and the upregulation of peripheral blood neutrophils in turn activates the immune response. Lymphocytopenia, on the other hand, is the result of continuous migration and infiltration of peripheral lymphocytes in the inflamed synovium of RA under the action of chemokines, while increased early apoptotic markers in peripheral blood lymphocytes may initiate an apoptotic cascade response, leading to an increase in apoptosis of lymphocytes in RA patients.^[9]^ Previously, a meta-analysis of 13 studies on the neutrophil-to-lymphocyte ratio (NLR) found that NLR was significantly associated with RA.^[10]^ Therefore, peripheral blood NLR for RA has great potential for exploration and analysis.

Current international standards of practice recommend the use of patient-reported outcomes (PROs), which have advantages over traditional physician-reported outcome metrics in disease surveillance, clinical efficacy assessment, and chronic disease management.^[11,12]^ Self-perception of patients (SPP), which refers to RA patients’ own responses to sensory source stimuli, is a core marker of the presence of the patient’s vital organism, and it encompasses PROs across a number of dimensions, including overall functioning, somatic pain, disease activity, and health-related quality of life.^[13,14]^ The International Consortium for Health Outcome Measurement considers the SPP scale to be an important standard for evaluating the outcomes of patients with arthritis, including RA, and it is regularly updated and recommended for global implementation.^[15]^ In conclusion, the SPP is an important outcome indicator for measuring the disease status of RA patients, and its association with NLR needs to be further tested.

**Figure 1.**
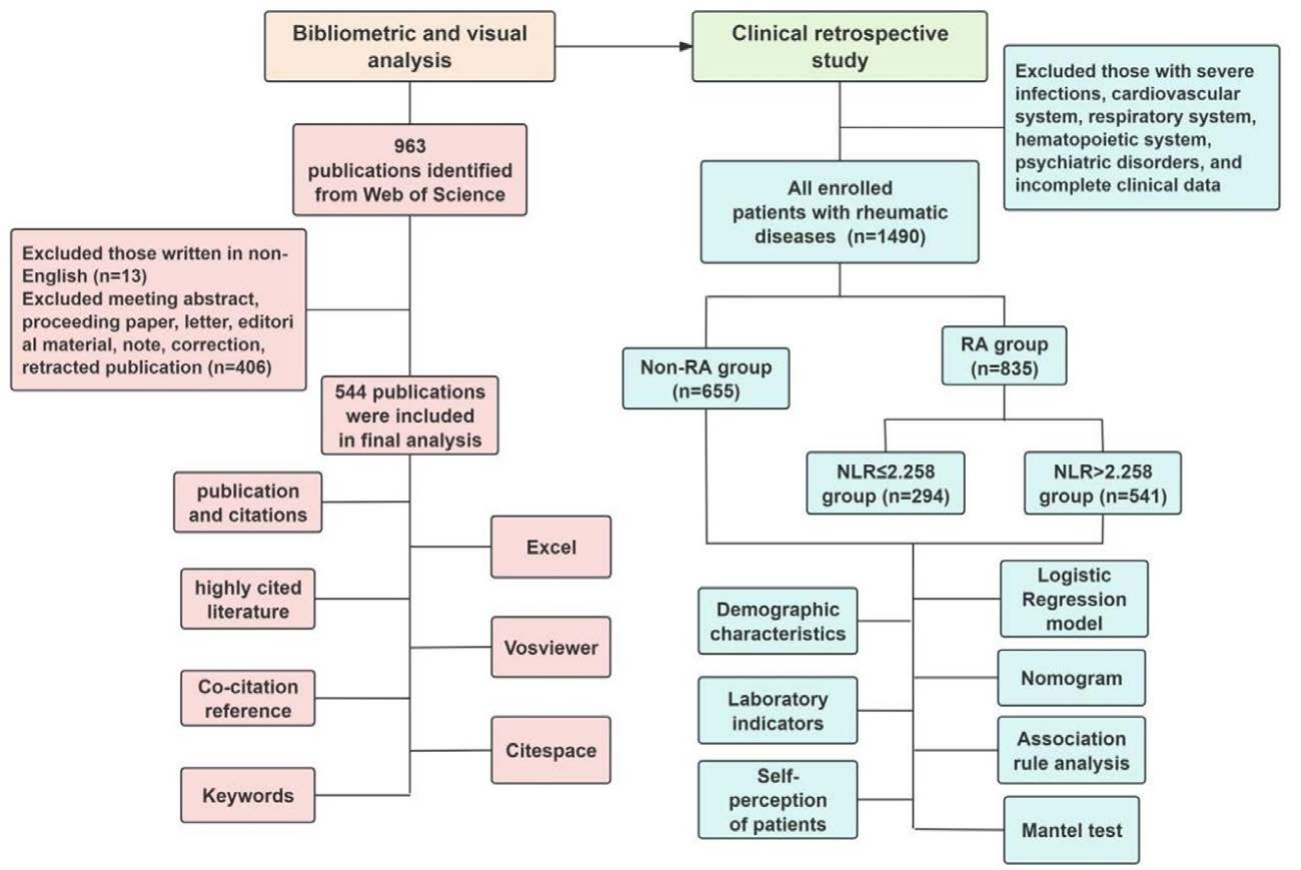
Flow chart of the study.

Literature visualization is a reliable statistical method for quantitative and qualitative intuitive analysis of the vast scientific literature related to a specific topic.^[16]^ We first attempted to use literature visualization techniques to systematically analyze the literature related to RA-NLR research published in the last 30 years in order to obtain the research base and hot topics in the field. Subsequently, from a clinical perspective and with the help of the platform of the First Affiliated Hospital of Anhui University of Chinese Medicine, we developed a nomogram to validate the predictive value of NLR in identifying the risk of RA, and built multiple logistic regression models to explore the relationship between NLR as a composite indicator of inflammation and high levels of somatic pain and disease activity in RA patients. Association rule analysis and the Mantel test identified correlations between NLR and RA clinical characteristics and reported outcomes. In addition, subgroup analyses were performed to verify the reliability of our findings. The specific flow of the study is shown in Figure 1.

## 2. Materials and methods

### 2.1. Literature source

This study referred to other similar studies.^[17]^ The Web of Science Core Collection (WoSCC) database from Clarivate Analytics covers more than 15,000 peer-reviewed journals published in more than 250 disciplines worldwide and is one of the most comprehensive and authoritative database platforms for global academic information.^[18]^ Therefore, we chose the SCI-Expanded (1900-present) database in WOSCC as the data source for literature retrieval.

### 2.2. Data acquisition and retrieval strategies

The literature search was completed in 1 day to allow for the bias caused by the rapid update of the database, and the last search date was March 1, 2024. In order to search all literatures related to NLR in RA extensively and accurately, we have adopted the search strategy as follows: TI= (“neutrophil” OR “neutrophils” OR “lymphocyte” OR “lymphocytes” OR “neutrophils/lymphocytes” OR “neutrophil-lymphocyte ratio” OR “neutrophil to lymphocyte ratio” OR “NLR”) AND TI = (“rheumatoid arthritis”), the search year is limited to January 1, 1994, to March 1, 2024. Language is limited to English. Article types are selected as article and review article, while meeting abstract, proceeding paper, letter, editorial material, note, correction and retracted publication are excluded from this study. A total of 544 literatures obtained as the final data set.

### 2.3. Literature visualization analysis

Literature visualization analysis mainly adopts VOSviewer, CiteSpace, and Microsoft Excel software. We used Microsoft Excel 2021 to plot global annual publication and citation trends and to create a time curve of the number of publications that can predict future trends. VOSviewer (version 1.6.18), a classic literature visualization software,^[19]^ extracts important parameters from a large number of scientific publications and is used to construct publication co-citation relationships and coexistence networks of keyword clusters. To assist in the visual review of research trends and emerging hotspots, CiteSpace (version 6.3.R1)^[20]^ was utilized for cluster analysis, timeline views, and burst mapping. The CiteSpace parameters were set as follows: the time span was set to January 1994 to March 2024. Select “Years per slice” as “1” and “k” for g-index as 10 or 5 to simplify the network architecture and draw attention to key components.

### 2.4. Clinical patients

This study collected rheumatology patients admitted to the Department of Rheumatology and Immunology of the First Affiliated Hospital of Anhui University of Chinese Medicine from May 2019 to December 2022. The hospital’s health information system contained basic information about all patients, such as age, gender, height, weight, disease duration, smoking and alcohol consumption. Further screening criteria excluded those patients with severe infections, severe cardiovascular, respiratory, hematopoietic disorders, psychiatric disorders, and incomplete clinical information. Ultimately, a total of 1490 rheumatologic patients participated in this study. This was a single-center, retrospective and observational study (Clinical trial number: not applicable), and all study procedures complied with the ethical requirements outlined in the Declaration of Helsinki and were approved by the Ethics Committee of the First Affiliated Hospital of Anhui University of Chinese Medicine (No. 2022MCZQ01).

### 2.5. Clinical indicators collection

In addition to the demographic characteristics of the patients, we collected clinical data of the patients during hospitalization, including routine blood test results and biochemical results. These were as follows: neutrophil count, lymphocyte count, ESR, C-reactive protein (CRP), RF, anti-cyclic citrullinated peptide antibody (anti-CCP), immunoglobulin A (IgA), immunoglobulin G (IgG), immunoglobulin M (IgM), complement component 3 (C3), complement component 4 (C4). The NLR index was calculated using the following formula: NLR = neutrophil count (10^9^/L)/lymphocyte count (10^9^/L).

### 2.6. Self-perception of patients

Clinical outcomes were assessed using SPP as our PROs for RA, including the Short Form 36 Health Survey (SF-36), Visual Analog Scale (VAS), Patient and Physician Globally Assessed Disease Activity based on the VAS (PGA, PhGA), Chinese Patient Reported Activity Index for Rheumatoid Arthritis Scale (CPRI-RA). The SF-36 is widely recommended as an important reference tool for evaluating physical and mental health (MH) in clinical studies of RA,^[21,22]^ including physical function (PF), role physical, body pain (BP), general health (GH), vitality (VT), social functioning (SF), and physical activity, SF, role emotional, MH, and health transformation. The SF-36 uses a score of 0 to 100 as a GH rating index, where equal to or >50 falls within the normal range. The VAS scale is a self-visualized intuitive pain symptom rating based on the results of self-visualization,^[23,24]^ and the scoring criteria are as follows: no pain is 0, mild pain is 1 to 3, moderate pain as 4 to 6, and severe pain as 7 to 10. CPRI-RA is a RA-PRO scale that evaluates the degree of disease activity of patients from the perspective of Chinese RA patients, providing an objective and quantitative complementary tool for the efficacy evaluation system of RA clinical trials.^[25,26]^ Anxiety self-assessment scale (SAS) and depression self-assessment scale (SDS) are important complementary tools for evaluating the psychological status of RA patients, which are worldwide widely promoted.^[27]^ Their standardized scores have a cutoff value of 50, with 50 to 59 being mild, 60 to 69 being moderate, and 70 or more being severe. All the scales were filled in under the expert supervision of the clinical laboratory staff at our hospital, with another physician responsible for supervision and quality control.

### 2.7. Logistic regression analysis

Univariate and multivariate logistic regression analyses were used to assess the independent predictors of RA. Meanwhile, 4 progressive multivariate logistic regression models were fitted to assess the association between high NLR levels and significant somatic pain and high disease activity. In model 1, crude ORs and their corresponding 95% CIs were calculated. In model 2, we adjusted for participants’ ESR and CRP. Model 3, adjusted for all immune and inflammatory markers. Model 4, with additional adjustments for age, gender, BMI, smoking status, and drinking status, on top of the adjustments in Model 3. In addition, we performed subcomponent stratification analyses to determine the stability of the effects of NLR on VAS, CPRI-RA.

### 2.8. Construction and evaluation of the nomogram

Based on the independent predictor variables, nomogram was developed using the “rms” and “regplot” packages in R. The “nomogramFormula,” “pROC,” and “rmda” packages were used to perform recipient operating characteristic (ROC) curves, calibration plots, and decision curve analysis (DCA) to assess performance of the model.

### 2.9. Random Forest algorithm

The Random Forest algorithm for regression is a suitable machine learning method for constructing a decision tree on a random subset of data and features in order to synthesize the significance of the predicted model variables while evaluating the error without restriction. The “randomForest” package in the R software is used to perform this process.

### 2.10. Association rule analysis

Association rule analysis based on the Apriori algorithm were used to determine the association between NLR and clinical characteristics of RA patients. We used elevated levels of NLR after treatment as the antecedent, while clinical indicators and SPP scores were the posterior, respectively. The minimum support level was set to 10%, the minimum confidence level was 60%, and the degree of improvement was > 1.

### 2.11. Mantel test

Mantel test, a test for determining the correlation between 2 distance measurement matrices, is performed by using the “linkET” and “ggplot2” packages in R were executed to explore the correlation between NLR and clinical features of RA.

### 2.12. Statistical analysis

All statistical analyses were performed via IBM SPSS Statistics 26.0. For continuous variable data, those with non-normal distributions were described as median and interquartile range and compared using the Mann–Whitney U test. Categorical variables were then described as frequencies and percentages and compared using the chi-square test. Two-tailed values of *P* ≤ .05 were considered statistically significant differences.

## 3. Results

### 3.1. Trends in global publications and citations

**Figure 2.**
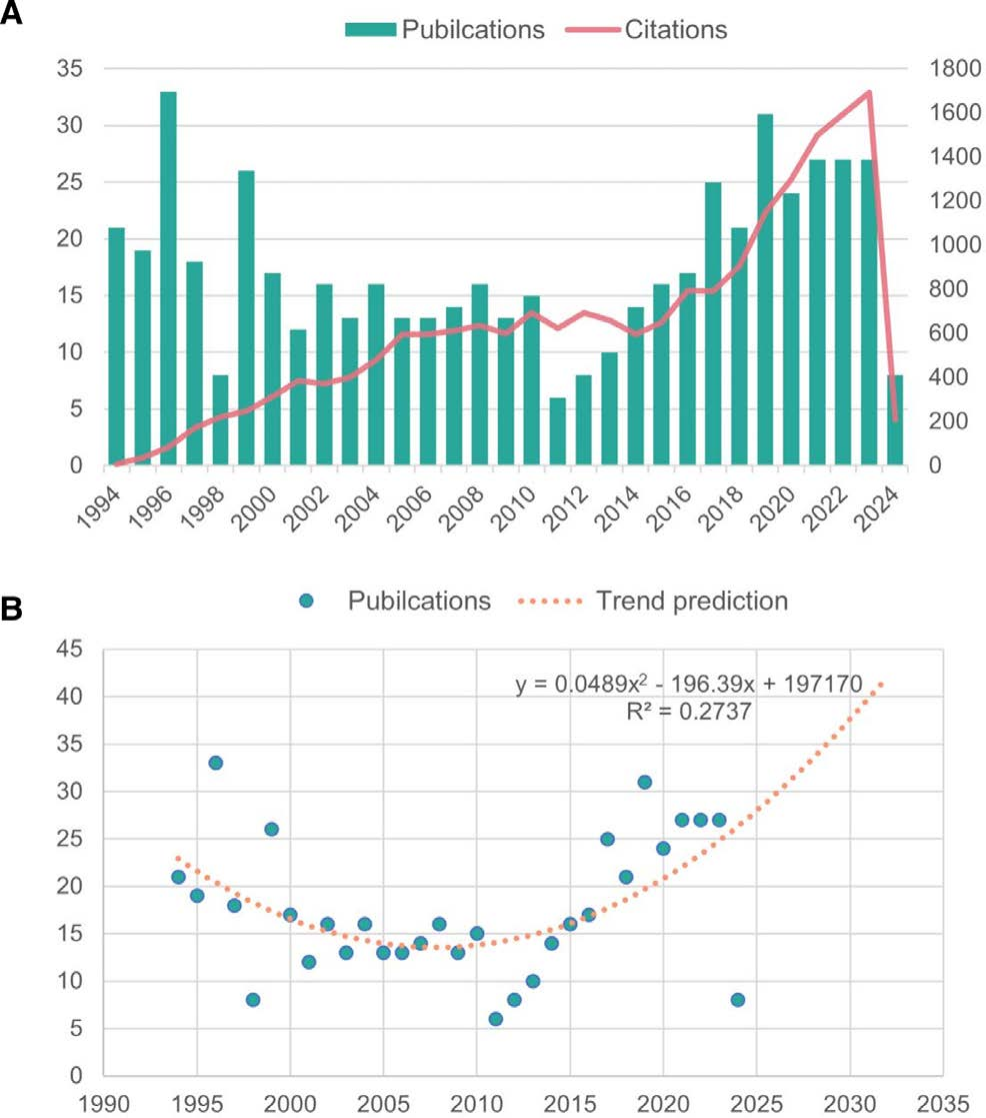
Trends in global publications and citations. (A) The annual number of publications and citations. (B) Fitting curve of overall annual growth trends in publications.

A total of 544 publications matched the inclusion criteria for the study through de-duplication sorting in CiteSpace, including 511 articles (93.93%) and 33 review articles (6.07%). The number of publications and citations over a specific period of time can objectively and quantitatively reflect the overall development trend of research in a certain field. The annual circulation and citations of publications in the RA-NLR field are shown in Figure 2A. Despite the fluctuating fallback in some years, the annual number of publications in the field shows an overall upward trend and stabilizes at more than 25 articles since 2021. Due to the high number of articles published during the plateau period, a new research explosion is yet to come. Based on our fitted curve prediction (Fig. 2B), the cumulative number of global publications in this field will continue to climb in the next decade, which may lead to a new breakthrough. Meanwhile, the citation frequency of these publications also shows an increasing trend year by year, with a total of 19,595 citations (18,613 times after self-citation exclusion), an average of 36.02 citations, the highest citation frequency of 487 times for a single article, and a total h-index of 69. These data indicate that the research on RA-NLR has attracted extensive attention from scholars and has shown a vigorous development trend in recent years, with a solid research foundation.

### 3.2. Analysis of highly cited literature

Subsequently, we launched an in-depth analysis of the highly cited literature. Of the 544 publications, 113 reached the threshold of a minimum number of citations ≥ 50. Table S1 (Supplemental Digital Content, https://links.lww.com/MD/Q66) summarizes the details of the top 20 most frequently cited publications on RA-NLR, with a citation frequency of 147 to 487. Only 3 of them were systematic reviews, and the rest were original studies. Among them, Wright et al^[28]^ published a review article entitled “The multifactorial role of neutrophils in rheumatoid arthritis” in Nature Reviews Rheumatology (IF = 33.7, 2023) ranked fourth with 373 citations, in which the authors comprehensively reviewed the multifactorial role of neutrophils in contributing to the pathology of RA through the release of cytotoxic and immunoregulatory molecules. The study by Carmona-Rivera et al^[29]^ published in Science Immunology (IF = 24.8, 2023) further explored the inter-crosstalk between synovial fibroblasts (FLS) and neutrophils in RA patients and mouse models, proposing that FLS can be activated by neutrophil extracellular traps (NETs) and presented to the adaptive immune system through internalization of citrullinated peptides, leading to RA pathogenic autoimmunity and cartilage damage. A study by Lundy et al^[30]^ reviewed data on genetics, animal models of arthritis, and cell-to-cell interactions and proposed that disruption of T-cell homeostasis can dramatically drive synovial inflammation and joint destruction. In addition, a 2015 study by Uslu et al,^[31]^ which included 104 RA patients and 51 healthy subjects, proposed NLR and PLR as new inflammatory markers that could be used to assess disease activity in RA patients. In conclusion, these highly cited papers were published in a relatively early year and have had a fundamental and seminal impact on the study of NLR in RA, triggering more research thinking for the field.

### 3.3. Co-cited reference analysis

**Figure 3.**
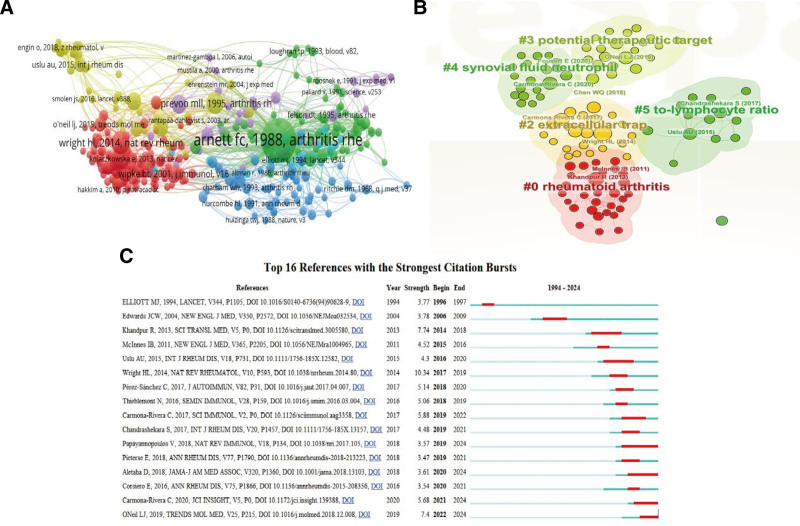
Co-cited reference analysis. (A) A map of 360 references with a minimum of 5 citations. (B) Cluster analysis map based on co-cited references for CiteSpace software analysis. (C) The top 16 references with strong citation burst, where blue lines represented time intervals and red lines stood for strong reference citation burst.

Co-cited reference is literature that has been co-cited by multiple publications and can therefore be considered as the knowledge structure and research base of the field. According to statistics, 15,139 relevant references were cited in 544 publications. Figure 3A shows the knowledge graph of highly co-cited references (with a minimum co-citation count of 5), consisting of 4 modules, 360 nodes, and 11,991 links. Table S2 (Supplemental Digital Content, https://links.lww.com/MD/Q66) lists the top 10 co-cited references, all of which have been cited at least 514 times. Subsequently, we performed a CiteSpace software-based cluster analysis to segment the reference literature into 5 main modules (Fig. 3B). Cluster 1 was RA (29 articles), with topics on the concept, diagnosis, and classification criteria of RA; cluster 2 was extracellular trap (23 articles), focusing on exploring the immune mechanisms and pathological responses related to NETs in RA; cluster 3 was potential therapeutic target (21 articles), focusing on the development and experimentation of potential therapeutic targets in RA; cluster 4, synovial fluid neutrophil (19 articles), focusing on exploring the receptors, proteins and pathways of action that interact with neutrophils in the local microenvironment of RA; cluster 5, to-lymphocyte ratio (13 articles). The research topics were related to clinical trials, diagnostic and therapeutic markers, and so on. In addition, citation bursts can reflect the references that are frequently cited by researchers in a particular field during a certain period of time. Figure 3C presents specific information about the top 16 references with strong citation bursts. While most of the reference bursts have ended, 4 references are still ongoing today. It is worth mentioning that 2 references address the importance and value of NLR as a new clinical indicator of inflammation in RA. One of them, published by Chandrashekara et al^[32]^ in 2017, entitled “Characterization of neutrophil-to-lymphocyte ratio as a measure of inflammation in rheumatoid arthritis,” which was clearly stated that the diagnostic efficacy of NLR is comparable to that of CRP and is independent of cytokines affecting CRP and ESR. In conclusion, the above highly co-cited references related to RA-NLR from multiple perspectives, mainly including the pathogenesis of RA and the study of inflammatory cellular responses.

### 3.4. Keyword co-occurrence, cluster, and burst analysis

**Figure 4.**
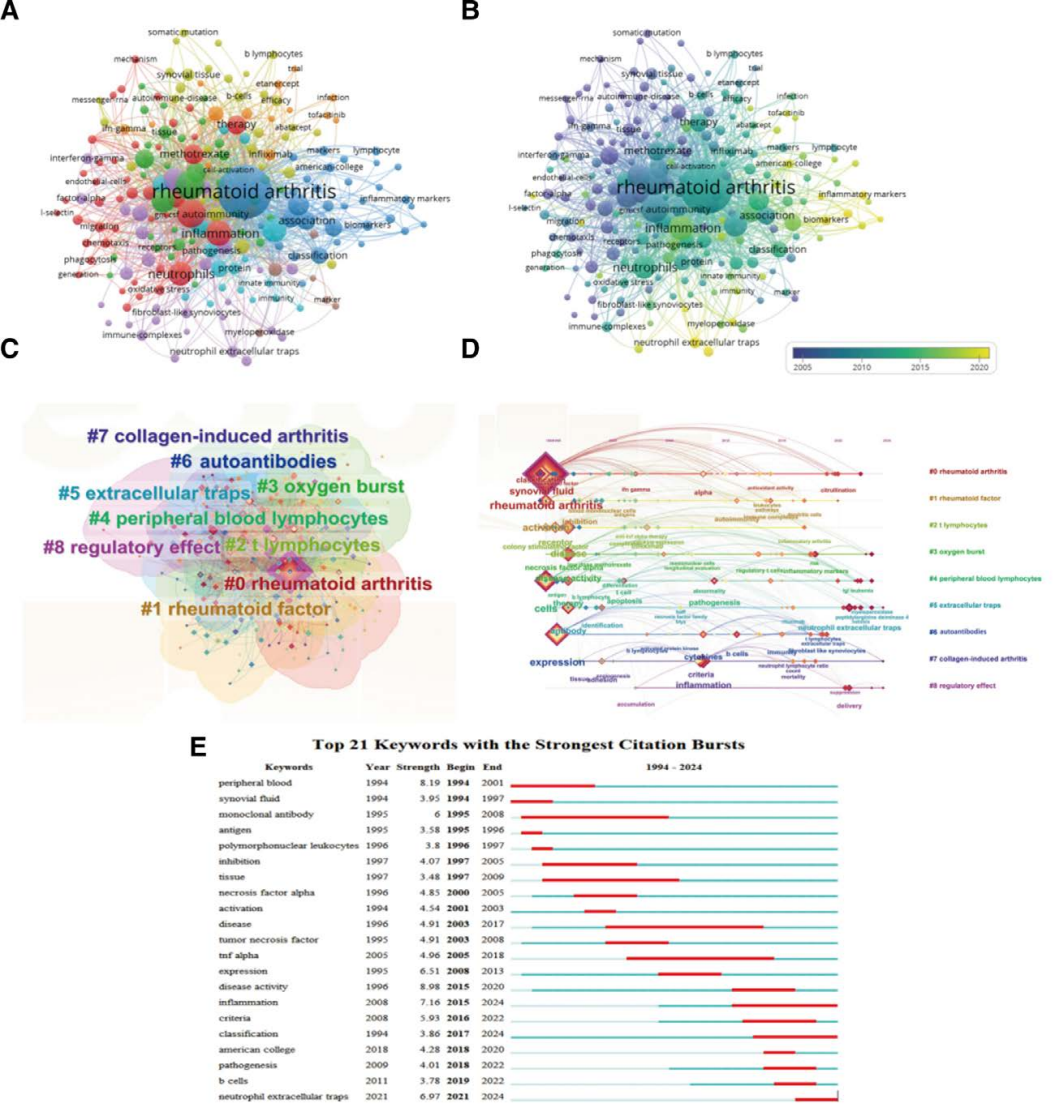
Keyword co-occurrence, cluster, and burst analysis. (A) The keyword co-occurrence network in studies on RA-NLR, where the size of the points represented the frequency. (B) Time network of keywords in publications on RA-NLR. (C) Keywords clustering analysis, where the keywords were divided into 9 groups. (D) Keywords clustering timeline view. (E) Analysis of the top 21 strongest burst keywords. NLR = neutrophil-to-lymphocyte ratio, RA = rheumatoid arthritis.

In addition, co-occurrence, clustering and burst analysis of keywords also help to discover the research themes and hot trends of publications. In VOSviewer, we frequency-ranked 2416 keywords and selected the keywords with co-occurrence number >5, and finally obtained a co-occurrence network consisting of 8 clusters, 207 nodes and 4383 connections (Fig. 4A). After removing repetitive or meaningless words, the top 10 high-frequency keywords were “inflammation” (68), “synovial-fluid” (59), “ activation” (57), “disease-activity” (47), “therapy” (39), “ peripheral-blood” (38), “tnf-alpha” (33), “criteria” (32), “ classification” (30), and “autoantibodies” (20). By marking the above high-frequency keywords with a specific color based on the average time of occurrence, it was found that “inflammatory markers” and “biomarkers” were widely noticed (Fig. 4B). Further, we categorized the 2416 keywords into 9 classes by cluster analysis, which were “rheumatoid arthritis,” “rheumatoid factor,” “ t lymphocytes,” “oxygen burst,” “peripheral blood lymphocytes,” “extracellular traps,” “autoantibodies,” “collagen-induced arthritis,” and “regulatory effect” (Fig. 4C). The timeline view of the keyword clustering shows the trend of the keywords over time and the relationship between different clustering groups. By plotting the timeline view, we found that the clusters of “autoantibodies,” “collagen-induced arthritis,” and “regulatory effect” clusters appeared later, suggesting that they are still the thematic direction of current research (Fig. 4D). In addition, we also performed the top 21 strongest keyword bursts (Fig. 4E). Among them, “disease activity” had the strongest outbreak, while keywords such as “inflammation” and “classification” were still continuing to explode. The above results indicate that NLR, as a relatively new inflammation indicator, deserves further study for its correlation with RA inflammation markers and disease activity.

### 3.5. Clinical characteristics of the study population

A total of 1490 patients with rheumatic diseases were recruited in our study and assigned to RA (n = 835) and non-RA groups (n = 655). The non-RA group contained 427 patients with osteoarthritis, 215 patients with ankylosing spondylitis and 13 patients with connective tissue disease. The demographic characteristics and laboratory indicators of the included population are shown in Table 1. we found significant differences between the 2 groups in terms of age, gender composition, course of disease, smoking and drinking (*P* < .05). Specifically, the RA group included 691 females and 144 males, ranging in age from 14 to 87 years. While the non-RA group included 407 females and 248 males, ranging in age from 13 to 89 years old. Patients in the RA group had a much higher age and course of disease than those in the non-RA group (*P* < .001), while BMI, smokers, and alcohol drinkers were relatively lower (*P* < .001 or *P* < .05). In addition, we observed that individuals with RA had significantly increased neutrophil counts and NLR levels but decreased lymphocyte counts compared to patients in the non-RA group (*P* < .001). Notably, in terms of ESR and CRP, RA patients also showed significantly higher levels (*P* < .001).

**Table 1:** Demographic and clinical characteristics of study groups.

Variables	RA group (n = 835)	Non-RA (n = 655)	*P* value
*Demographic characteristics*			
Gender, n (%)			
Male	144 (17.25%)	248 (37.86%)	<.001
Female	691 (82.75%)	407 (62.13%)	
Age (yr)	57.00 (51.00, 68.00)	54.00 (42.00, 64.00)	<.001
BMI (kg/m^2^)	22.07 (20.55, 23.83)	23.46 (21.46, 25.83)	<.001
Course of disease (yr)	9.53 (3.00, 15.00)	6.00 (2.00, 10.00)	<.001
Smoking, n (%)	38 (4.55%)	46 (7.02%)	.040
Drinking, n (%)	25 (2.99%)	35 (5.34%)	.022
*Laboratory indicators*			
Neutrophil count (×10^9^)	4.06 (2.96, 5.56)	3.20 (2.48, 4.07)	<.001
Lymphocyte count (×10^9^)	1.43 (1.07, 1.81)	1.78 (1.4, 2.19)	<.001
NLR	2.83 (1.89, 4.13)	1.76 (1.36, 2.25)	<.001
ESR (mm/h)	34.00 (17.00, 59.00)	11.00 (4.00, 22.00)	<.001
CRP (mg/L)	12.00 (3.37, 35.85)	1.66 (0.58, 7.48)	<.001

BMI = body mass index, CRP = C-reactive protein, ESR = erythrocyte sedimentation rate, NLR = neutrophil-to-lymphocyte ratio, RA = rheumatoid arthritis.

### 3.6. Exploration and construction of the RA risk prediction model

**Figure 5.**
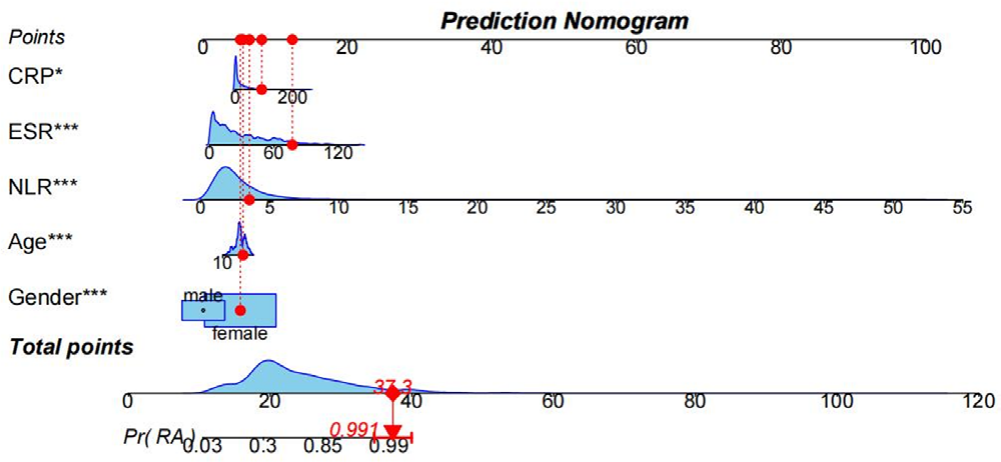
Nomograms estimate the risk of the RA occurrence. Depending on the individual, the values of the predictor variables were first determined on each axis, and a vertical line was plotted upwards to the “Point” axis to determine the corresponding scores obtained by the patient. Then, the scores of all factors were summed and the total value is determined on the “Total points” line. Finally, a vertical line is drawn to measure the probability of the patient having RA. For example, the factors female (5 points), age 63 years (6.78 points), NLR = 3.53 (6.35 points), ESR = 77 (11.55 points), and CRP = 92.13 (7.69 points) totaled approximately 37.3 points, which is estimated to be a 99.1% probability of developing RA. CRP = C-reactive protein, ESR = erythrocyte sedimentation rate, NLR = neutrophil-to-lymphocyte ratio, RA = rheumatoid arthritis.

Subsequently, we constructed univariate and multivariate logistic regression models to explore the independent predictors of RA, respectively (Table 2). After adjusting for all confounders in the univariate model, we found that gender, age, NLR, ESR, and CRP were independently associated with RA, and consequently, identified them as independent predictors of RA. To this end, we developed a nomogram to further explain the contribution and significance of the above clinical predictor variables. We assigned a line to each predictor variable, with the length of the line segment reflecting the magnitude of the factor’s contribution to the event of RA occurrence. At the bottom of the column-line graph is a total score line, which is the sum of the individual scores corresponding to each variable at different individual values, and the resulting total score corresponds to the individual risk of RA occurrence (Fig. 5). The results revealed that NLR had the most superior degree of contribution, with a subsequent increase of 10 points in the model score for every 5 increase. Model scores increased by 5 points for female patients compared with males. 60-year-old patients corresponded to a 6-point increase in score. 9 points were added to the model score for each 60-mm/h increase in ESR. When CRP was 200 mg/L, the corresponding score increased by 12 points.

**Table 2:** Logistic regression models for RA risk prediction.

Variables	Crude model			Adjusted Model		
	OR	95% CI	*P* value	OR	95% CI	*P* value
Gender (male Ref.)	2.924	2.302–3.714	<.001	5.210	3.599–7.541	<.001
Age	1.029	1.021–1.037	<.001	1.014	1.004–1.024	.006
BMI	0.901	0.871–0.933	<.001	0.960	0.920–1.001	.057
Course of disease	1.035	1.022–1.049	<.001	1.015	0.998–1.031	.077
Smoking	0.631	0.406–0.982	.042	1.586	0.784–3.205	.199
Drinking	0.547	0.324–0.923	.024	1.564	0.683–3.579	.290
Neutrophil count	1.448	1.35–1.553	<.001	1.046	0.852–1.285	.666
Lymphocyte count	0.409	0.341–0.491	<.001	0.793	0.511–1.230	.300
NLR	2.018	1.815–2.244	<.001	1.632	1.211–2.200	.001
ESR	1.057	1.050–1.064	<.001	1.047	1.037–1.056	<.001
CRP	1.049	1.040–1.058	<.001	1.012	1.001–1.024	.028

Crude model: unadjusted. adjusted model: adjusted for all inclusion factors including gender, age, BMI, course of disease, smoking, drinking, neutrophil count, lymphocyte count, NLR, ESR, and CRP.

BMI = body mass index, CRP = C-reactive protein, ESR = erythrocyte sedimentation rate, NLR = neutrophil-to-lymphocyte ratio, OR = odd ratio, RA = rheumatoid arthritis.

### 3.7. Evaluation and validation of the nomogram of the RA prediction model

**Figure 6.**
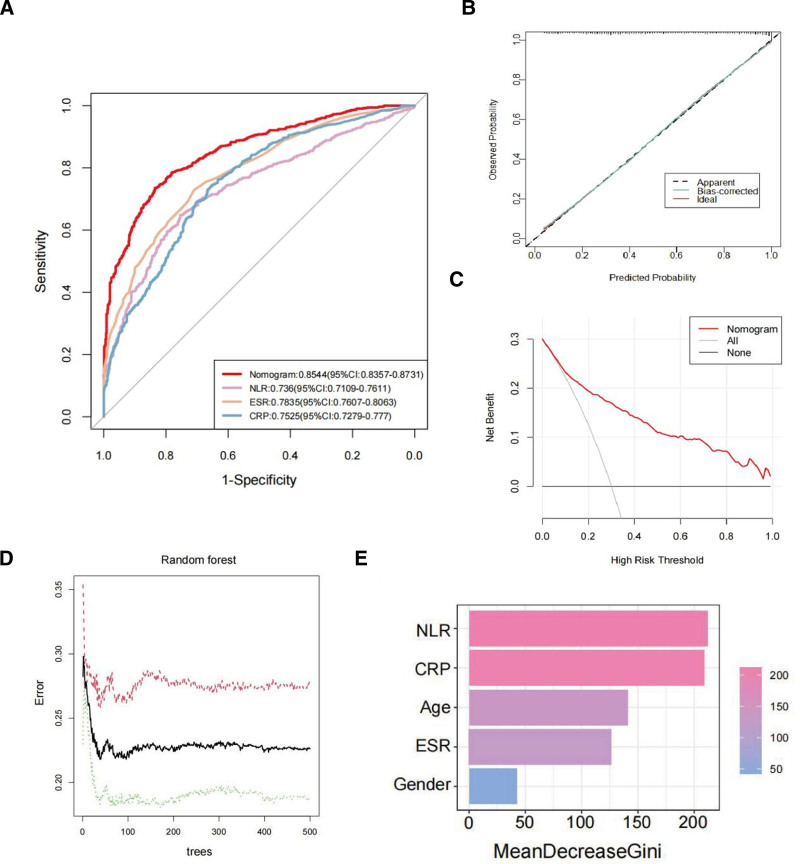
Evaluation and validation of the nomogram of the RA prediction model. (A) The ROC curves for nomogram model, NLR, ESR, and CRP diagnostic performance. (B) The calibration curve of the nomogram model. (C) DCA for the nomogram model. (D) Randomized forest tree diagram, where the x-axis represents the number of trees, the y-axis represents the error rate, and the red, green, and black points represent RA samples, non-RA samples, and all samples, respectively. (E) The Random Forest algorithm scores and ranks meaningful variables within the nomogram model. CRP = C-reactive protein, DCA = decision curve analysis, ESR = erythrocyte sedimentation rate, NLR = neutrophil-to-lymphocyte ratio, RA = rheumatoid arthritis, ROC = recipient operating characteristic.

To further explore the performance of the nomogram model and key predictor variable, we plotted the ROC curve, calibration curve, and DAC curve, respectively. For the RA prediction nomogram model, the area under the curve (AUC) of the model was 0.799 (95% CI: 0.752–0.846), suggesting that the model has high discrimination in RA (Fig. 6A). Some parameters related to the diagnostic performance of NLR, ESR and CRP as predictor variables of RA as well as the best cutoff values are presented detailedly in Table 3. Overall, ESR had the best predictive performance (AUC = 0.7835), followed by CRP (AUC = 0.7525). We found that when the best cutoff value for NLR was 2.258, the AUC was 0.736, the diagnostic sensitivity was 64.79%, the specificity was 75.42%, and the accuracy was 69.46%. These results indicated that NLR, ESR and CRP all had satisfactory diagnostic efficacy (AUC all >0.7), especially NLR performed well in diagnostic specificity. The calibration curves showed that the simulated curves basically overlapped with the actual curve trend trajectories, indicating a high degree of consistency and high calibration of the model (Fig. 6B). To assess the clinical utility of the column-line diagram, we also plotted the DCA curve (Fig. 6C). We found that when the threshold probability was >10%, the net benefit of using RA to predict the column-line diagram exceeded the thin gray line (all clinical outcomes occurred) and the black line (all clinical outcomes did not occur), indicating high clinical utility. Furthermore, we further rank the importance of the all variables within the model by the Random Forest algorithm (Fig. 6D and E). Encouragingly, the NLR represented the highest significance, followed by CRP. Overall, these results highlighted the unique and superior value of the nomogram model and especially the NLR in RA diagnosis.

**Table 3:** Assessment of the ability of NLR, ESR, and CRP in the diagnosis of RA

Variables	AUC	Cutoff value	Sensitivity (%)	Specificity (%)	Accuracy (%)	Maximum Youden index	PPV (%)	NPV (%)	95% CI
NLR	0.7360	2.258	64.79	75.42	69.46	0.402	77.07	62.69	0.7109–0.7611
ESR	0.7835	18.500	72.81	70.99	72.01	0.438	76.19	67.20	0.7607–0.8063
CRP	0.7525	3.555	74.49	65.19	70.40	0.397	73.18	66.72	0.7279–0.7770

95% CI = 95% confidence interval, AUC = the area under the curve, BMI = body mass index, CRP = C-reactive protein, ESR = erythrocyte sedimentation rate, NLR = neutrophil-to-lymphocyte ratio, NPV = negative predictive value, PPV = positive predictive value, RA = rheumatoid arthritis.

### 3.8. Differences in the clinicopathological features of RA between the NLR subgroups

To further understand the relationship between NLR and clinicopathologic characteristics of RA patients, we divided and compared 835 RA patients according to the NLR cutoff value (2.258) (Table 4). We observed that for baseline data, NLR showed significant differences in gender composition and age (*P* < .001 or *P* < .05), while there were no differences in BMI, course of disease, smoking and drinking. Secondly, among the laboratory indices, patients with NLR > 2.258 possessed higher ESR, CRP, RF and C3 levels and lower IgG levels (all, *P* < .01). The SPP score is an important outcome indicator for comprehensively evaluating disease activity and quality of life in RA patients. We found that patients with NLR > 2.258 exhibited higher levels of VAS, PGA, PhGA and CPRI-RA. These results suggest that RA patients with NLR > 2.258 may exhibit higher inflammatory response, severe somatic pain, and higher disease activity.

**Table 4:** Clinicopathological features of RA between the NLR subgroups

Variables	Total (n = 835)	NLR		*P* value
		>2.258 (n = 541)	≤2.258 (n = 294)	
*Demographic characteristics*				
Gender, n (%)				
Male	144 (17.25%)	114 (21.07%)	30 (10.20%)	
Female	691 (82.75%)	427 (78.93%)	264 (89.80%)	<.001
Age (yr)	57.00 (51.00, 68.00)	58.00 (51.00,68.00)	56.00 (51.00,66.00)	.032
BMI (kg/m^2^)	22.07 (20.55, 23.83)	22.21 (20.57, 23.92)	21.91 (20.45,23.72)	.560
Course of disease (yr)	9.53 (3.00, 15.00)	9.00 (3.00,14.00)	10.00 (4.00,15.77)	.195
Smoking, n (%)	38 (4.55%)	30 (5.55%)	8 (2.72%)	.061
Drinking, n (%)	25 (2.99%)	18 (3.33%)	7 (2.38%)	.444
*Laboratory indicators*				
ESR (mm/h)	34.00 (17.00, 59.00)	38.00 (19.00,62.00)	28.00 (13.00,50.00)	<.001
CRP (mg/L)	12.00 (3.37, 35.85)	17.88 (5.27,43.35)	5.23 (1.68,16.54)	<.001
RF (U/mL)	101.60 (38.90,253.30)	116.60 (40.65, 297.05)	77.10 (36.43,200.70)	.005
Anti-CCP (U/mL)	77.90 (15.30,213.00)	82.10 (16.10,207.50)	75.15 (13.15,233.75)	.627
IgA (g/L)	2.67 (1.99,3.64)	2.75 (2.04,3.64)	2.60 (1.86,3.66)	.150
IgG (g/L)	11.70 (9.30,14.57)	11.40 (8.98,14.44)	12.06 (9.89,14.95)	.009
IgM (g/L)	1.30 (0.97,1.73)	1.30 (0.95,1.75)	1.30 (1.00,1.72)	.756
C3 (g/L)	1.23 (1.08,1.37)	1.24 (1.12,1.39)	1.19 (1.03,1.33)	<.001
C4 (g/L)	0.30 (0.23,0.37)	0.30 (0.23,0.38)	0.29 (0.23,0.36)	.426
PF	25.00 (20.00,40.00)	25.00 (15.00,40.00)	30.00 (20.00,40.00)	.220
RP	0.00 (0.00,0.00)	0.00 (0.00,0.00)	0.00 (0.00,0.00)	.075
BP	31.00 (22.00,41.00)	31.00 (22.00,41.00)	31.00 (22.00,42.00)	.351
GH	30.00 (20.00,40.00)	30.00 (20.00,40.00)	33.50 (20.00,40.00)	.084
VT	40.00 (30.00,50.00)	40.00 (30.00,50.00)	40.00 (30.00,50.00)	.941
SF	50.00 (20.00,50.00)	50.00 (25.00,50.00)	50.00 (37.50,50.00)	.468
RE	0.00 (0.00,33.33)	0.00 (0.00,33.33)	0.00 (0.00,33.33)	.516
MH	48.00 (32.00,52.00)	44.00 (32.00,52.00)	48.00 (32.00,56.00)	.497
HT	75.00 (75.00,100.00)	75.00 (75.00,100.00)	75.00 (75.00,100.00)	.726
VAS (cm)	6.50 (5.60,7.30)	6.80 (6.00,7.50)	6.00 (5.00,7.00)	.007
PGA (cm)	6.30 (5.00,7.20)	6.70 (5.00,7.20)	6.00 (5.00,7.20)	.021
PhGA (cm)	6.00 (5.00,7.00)	6.00 (5.00,7.00)	6.00 (5.00,7.00)	.045
CPRI-RA	10.04 (8.43,11.60)	10.22 (8.67,11.60)	9.81 (8.17,11.31)	.007
SAS	55.00 (50,63.75.00)	55.00 (50.00,63.75)	55.00 (50.00,62.50)	.814
SDS	61.25 (56.25,67.50)	61.25 (56.25,67.50)	60.63 (55.00,66.25)	.233

Anti-CCP = anti-cyclic citrullinated peptide antibody, BMI = body mass index, BP = body pain, CPRI-RA = Chinese patient reported activity index for rheumatoid arthritis, CRP = C-reactive protein, ESR = erythrocyte sedimentation rate, GH = general health, HT = health transformation, MH = mental health, PF = physical function, PGA = patient assessment based on VAS, PhGA = Physician assessment based on VAS, RE = role emotional, RF = rheumatoid factor, RP = role physical, SAS = self-assessment anxiety scale, SDS = self-assessment depression scale, VAS = visual analog scale, VT = vitality.

### 3.9. Correlation between elevated NLR and laboratory indicators, SPP in RA patients

**Figure 7.**
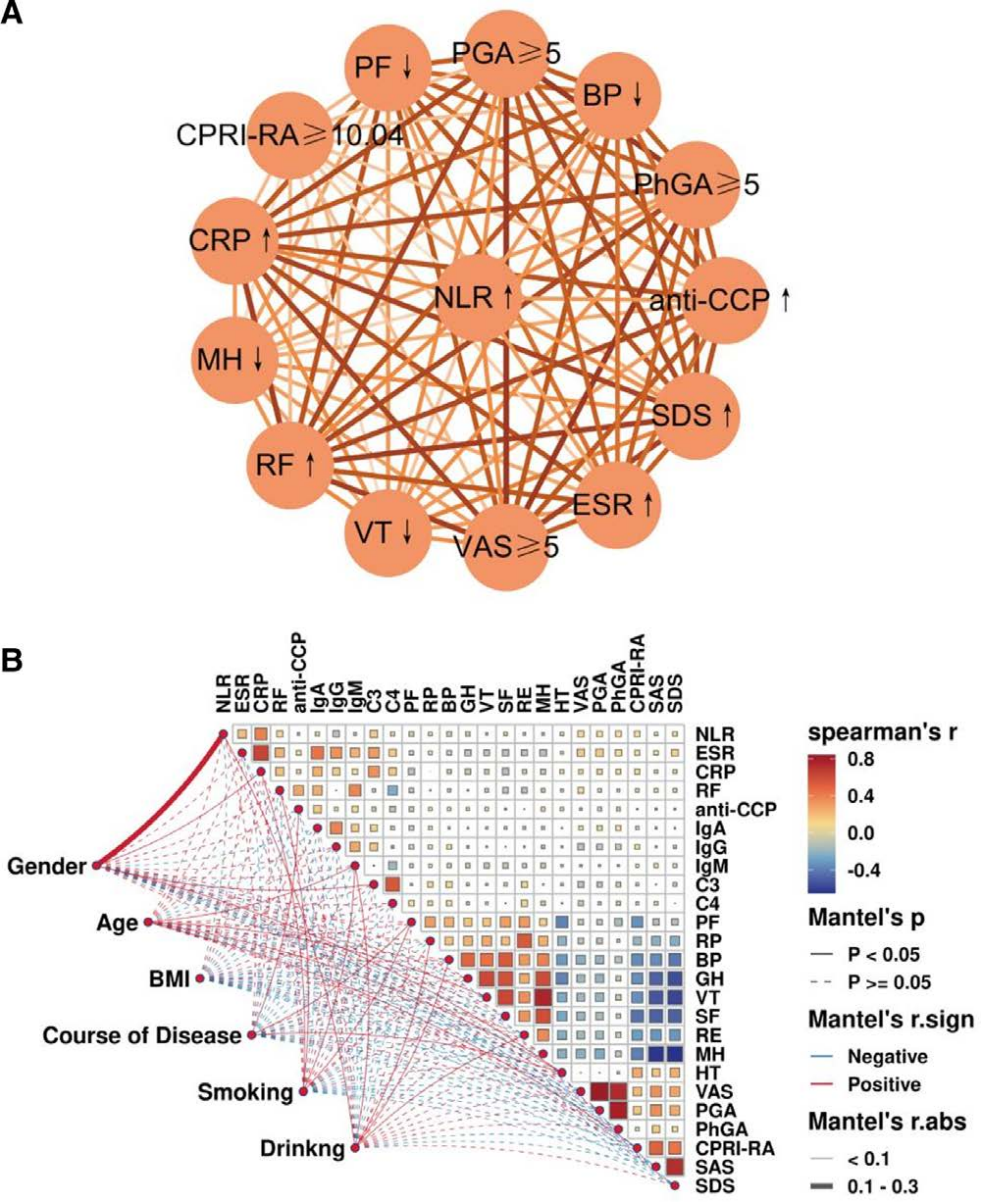
Correlation between elevated NLR and laboratory indicators, SPP in RA patients. (A) Association rules analysis for high NLR (defined as > 2.258) and other elevated laboratory indicators and abnormal SPP scores; (B) impact of gender, age, BMI, course of disease, smoking and drinking on baseline levels of laboratory indicators. BMI = body mass index, NLR = neutrophil-to-lymphocyte ratio, RA = rheumatoid arthritis, SPP = Self-perception of patients.

Further, we used association rule analysis to identify the correlation between higher NLR levels at baseline with laboratory indicators and SPP. We defined NLR > 2.258 as the antecedent, and elevated immuno-inflammatory indicators and abnormal SPP scores as the posterior. The results demonstrated that NLR > 2.258 was highly associated with abnormally elevated ESR, CRP, RF, anti-CCP, VAS ≥ 5, PGA ≥ 5, PhGA ≥ 5, and SDS, as well as a strong correlation with decreased levels of PF, BP, VT, and MH, and all of the above associations had a support level of >50%, confidence level of >60%, and a gain of >1 (Table 5 and Fig. 7A). In order to identify the role of the included factors on the laboratory indicators and the relationship between the indicators, we performed the Mantel test on the initial levels of the included laboratory indicators and the associated factors, and generated a correlation heat map matrix (Fig. 7B). Spearman correlation analysis found that the NLR had a strong correlation with ESR, CRP, RF, C3, VAS, PGA, PhGA, and CPRI-RA were significantly positively correlated (all, *P* < .01), whereas negatively correlated with IgG, PF, and BP levels (all, *P* < .05), and these results are shown in detail in Table S3 (Supplemental Digital Content, https://links.lww.com/MD/Q66). In addition, we also observed that gender was the most influential factor for NLR levels (*R* = 0.110, *P* = .001). In conclusion, the above results provide more evidence that NLR is closely associated with the clinic features of RA.

**Table 5:** Association rules analysis for high NLR and elevated laboratory indicators and abnormal SPP scores.

Items (Antecedent ⇒ Consequent)	Support (%)	Confidence (%)	Lift
NLR↑ ⇒ ESR↑	64.79	84.29	1.030
NLR↑ ⇒ CRP↑	64.79	95.01	1.049
NLR↑ ⇒ RF↑	64.79	92.98	1.006
NLR↑ ⇒ anti-CCP↑	64.79	85.40	1.010
NLR↑ ⇒ PF↓	64.79	83.73	1.009
NLR↑ ⇒ BP↓	64.79	83.55	1.011
NLR↑ ⇒ VT↓	64.79	65.62	1.004
NLR↑ ⇒ MH↓	64.79	61.18	1.006
NLR↑ ⇒ VAS ≥ 5	64.79	91.68	1.018
NLR↑ ⇒ PGA ≥ 5	64.79	90.02	1.019
NLR↑ ⇒ PhGA ≥ 5	64.79	89.46	1.018
NLR↑ ⇒ SDS↑	64.79	91.31	1.003

Up arrow indicates a downward adjustment; down arrow indicates a downward adjustment.

Anti-CCP = anti-cyclic citrullinated peptide antibody, BMI = body mass index, BP = body pain, CPRI-RA = Chinese patient reported activity index for rheumatoid arthritis, CRP = C-reactive protein, ESR = erythrocyte sedimentation rate, GH = general health, HT = health transformation, MH = mental health, NLR = neutrophil-to-lymphocyte ratio, PF = physical function, PGA = patient assessment based on VAS, PhGA = Physician assessment based on VAS, RE = role emotional, RF = rheumatoid factor, RP = role physical, SAS = self-assessment anxiety scale, SDS = self-assessment depression scale, VAS = visual analog scale, VT = vitality.

### 3.10. Predictive value of NLR in RA joint pain and disease activity status

Next, we built 4 logistic regression models adjusted for different factors to find out the role of NLR in RA joint pain and disease activity status, as shown in Table 6. We found that when the dependent variable was VAS score, NLR > 2.258 was always an independent predictor of moderate and severe pain even in Model 4, which adjusted for all registration parameters such as age, gender, BMI, course of disease, smoking, drinking, and various laboratory indicators (OR = 2.890, 95% CI = 1.101–7.589, *P* = .031; OR = 3.749, 95% CI = 1.416–9.923, *P* = .008). Similarly, NLR > 2.258 was a significant predictor of PGA ≥ 6.3 to all degrees (OR = 1.501, 95% CI = 1.104–2.041, *P* = .010). In addition, when the dependent variable was a high level of CPRA-RA score (defined as ≥ 9), NLR also demonstrated superior predictive utility, with ORs of 1.407, 1.509, 1.527, and 1.563 across the 4 models (all *P* < .05). Thus, we can determine that NLR is a key predictor for evaluating RA joint pain and disease activity status.

**Table 6:** Logistic regression model for NLR in RA joint pain and disease activity status.

	Model 1		Model 2		Model 3		Model 4	
	OR (95% CI)	*P* value	OR (95% CI)	*P* value	OR (95% CI)	*P* value	OR (95% CI)	*P* value
VAS (<4 Ref.)								
4–7	3.619 (1.446–9.061)	.006	3.179 (1.241–8.142)	.016	2.975 (1.146–7.725)	.025	2.890 (1.101–7.589)	.031
7–10	4.789 (1.901–12.061)	.001	4.192 (1.625–10.815)	.003	3.845 (1.468–10.067)	.006	3.749 (1.416–9.923)	.008
PGA (<6.3 Ref.)	1.490 (1.120–1.983)	.006	1.464 (1.090–1.966)	.011	1.462 (1.079–1.981)	.014	1.501 (1.104–2.041)	.010
PhGA (<6.3 Ref.)	1.224 (0.918–1.634)	.169	1.221 (0.907–1.643)	.188	1.213 (0.895–1.645)	.213	1.239 (0.911–1.685)	.173
CPRI-RA (<9 score Ref.)	1.470 (1.089–1.985)	.012	1.509 (1.108–2.054)	.009	1.527 (1.115–2.092)	.008	1.563 (1.135–2.152)	.006

Model 1: no adjustments were made; Model 2: adjustments were made for NLR (>2.258), ESR (>18.5), and CRP (>3.555); Model 3: adjustments were made based on what was in Model 2 and for other laboratory markers, which included RF, anti-CCP, IgA, IgG, IgM, C3, and C4; Model 4: adjustments were made for the Model 3 terms as well as age, gender, BMI, course of disease, smoking and drinking were adjusted.

95% CI = 95% confidence interval, anti-CCP = anti-cyclic citrullinated peptide antibody, BMI = body mass index, CPRI-RA = Chinese patient reported activity index for rheumatoid arthritis, CRP = C-reactive protein, ESR = erythrocyte sedimentation rate, IgA = immunoglobulin A, IgG = immunoglobulin G, IgM = immunoglobulin M, OR = odd ratio, PGA = patient assessment based on VAS, PhGA = Physician assessment based on VAS, VAS = visual analog scale.

### 3.11. Reliability of NLR in predicting VAS and CPRI-RA in different subgroups

**Figure 8.**
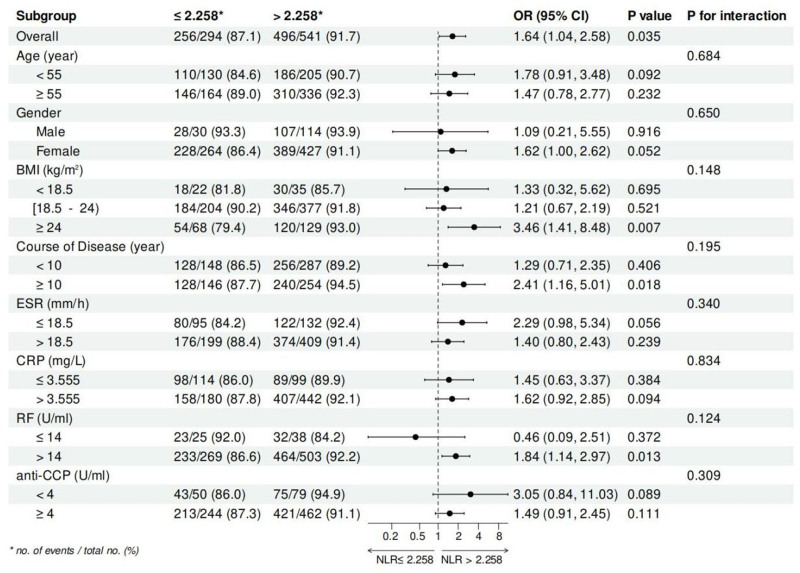
The reliability of high NLR (>2.258) in predicting the presence of severe somatic pain, as defined by high VAS scores (≥5 cm), was analyzed in subgroup stratification analysis. NLR = neutrophil-to-lymphocyte ratio, VAS = visual analog scale.

**Figure 9.**
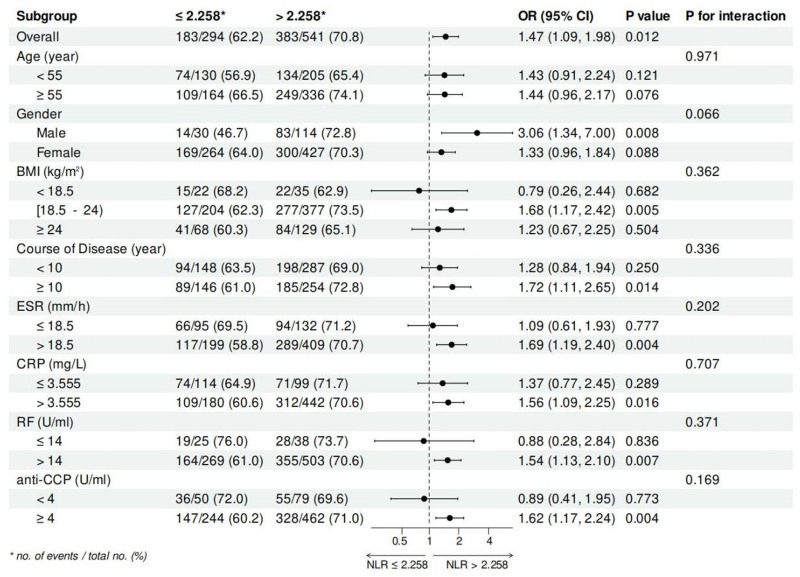
The reliability of high NLR (>2.258) in predicting the presence of severe somatic pain, as defined by high CPRI-RA scores (≥9), was analyzed in subgroup stratification analysis. CPRI-RA = Chinese patient reported activity index for rheumatoid arthritis, NLR = neutrophil-to-lymphocyte ratio.

To further evaluate the predictive performance of NLR in VAS and CPRI-RA, we performed subgroup stratification analysis (Fig. 8 and Fig. 9). When stratified by BMI, overweight patients (BMI ≥ 24) in the high NLR group (> 2.258) had a 3.46-fold (95% CI = 1.41–8.48, *P* = .007) higher risk of developing a VAS score of ≥ 5 than those in the low NLR group. For patients with a disease duration of ≥ 10 years, the risk of developing a high VAS score was 2.41 times higher in the high NLR group than in the low NLR group (95% CI = 1.16–5.01, *P* = .018). Among those patients with elevated RF values, higher levels of NLR increased the risk of developing a high VAS score by 0.84-fold (95% CI = 1.14–2.97, *P* = .013). In addition, gender, age, BMI, course of disease, ESR, CRP, RF, and anti-CCP did not change the predictive effect of NLR on high VAS scores in RA patients (all, p for interaction > 0.05). When the dependent variable was a high CPRI-RA score (≥ 9), male patients in the high NLR group had a 3.06-fold higher risk than those in the low NLR group (95% CI = 1.34–7.00, *P* = .008). For patients with BMI ≥ 18.5 and < 24, the OR for the high NLR group was 1.68 (95% CI = 1.17–2.42, *P* = .005). Among patients with a course of disease of ≥ 10 years, the risk of developing a high CPRI-RA score was 1.72 times higher in the high NLR group than in the low NLR group (95% CI = 1.11–2.65, *P* = .014). In those patients with high levels of ESR and CRP, the risk in the high NLR group was 1.69 times (95% CI = 1.19–2.40, *P* = .004) and 1.56 times (95% CI = 1.09–2.25, *P* = .016) that of the low NLR group. For patients with abnormally elevated RF and anti-CCP, high NLR then increased the risk by 0.54-fold (95% CI = 1.13–2.10, *P* = .007) and 0.62-fold (95% CI = 1.17–2.24, *P* = .004), respectively. In addition, these factors had no effect on the predictive effect of NLR on high CPRI-RA scores in patients with RA (all, P for interaction > 0.05).

### 3.12. Correlation of reduced NLR and remission in RA patients

**Figure 10.**
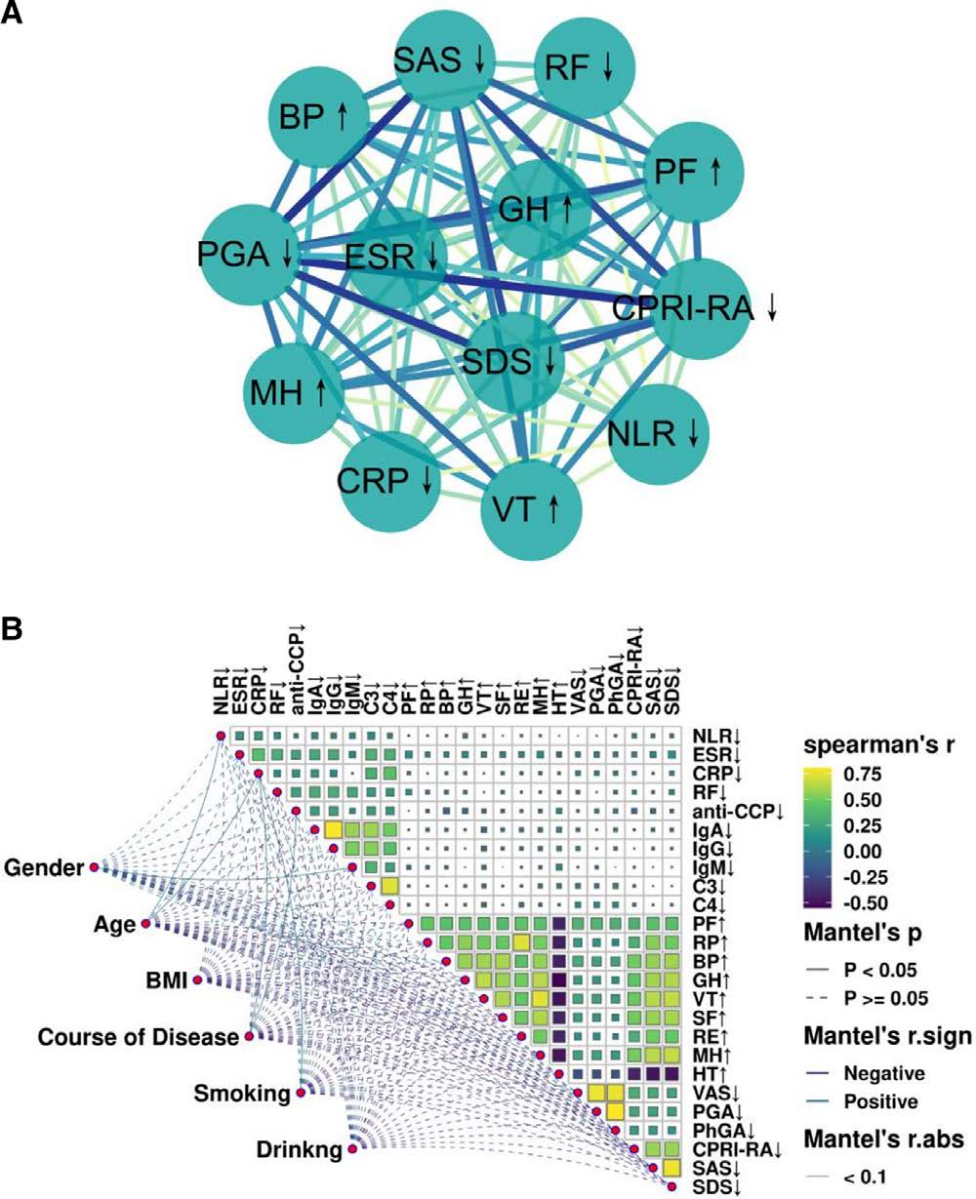
Correlation of reduced NLR and remission in RA patients. (A) Association rule analysis of reduction of NLR and reduction of immune inflammation index and recovery of SPP score after treatment; (B) Effect of gender, age, BMI, course of disease, smoking and drinking on laboratory indicators and SPP recovery. BMI = body mass index, NLR = neutrophil-to-lymphocyte ratio, RA = rheumatoid arthritis, SPP = self-perception of patients.

Finally, we assessed the correlation between NLR and remission of RA, which was mainly characterized by the reduction of immuno-inflammatory markers and the recovery of SPP scores. The results of the association rule analysis showed that the reduction in NLR levels was highly associated with remission of ESR, CRP, RF, PGA, CPRI-RA, SAS, and SDS, as well as a strong correlation with improvement in the levels of PF, BP, GH, VT, and MH, and that all associations had a support >40%, a confidence level >60%, and a gain >1 (Table S4, Supplemental Digital Content, https://links.lww.com/MD/Q66 and Fig. 10A). Further, the results of Spearman analysis further revealed that the decrease in NLR was positively correlated with the degree of reduction in ESR, CRP, RF, IgA, IgG, IgM, C3, and C4 metrics (all, *P* < .05), which are demonstrated in Table S5 (Supplemental Digital Content, https://links.lww.com/MD/Q66). Additionally, we also observed that the age contribution to the degree of remission of NLR by Mantel test was most significant (*R* = 0.055, *P* = .035) (Fig. 10B). These evidences suggest that NLR can reflect the degree of remission in RA patients and can be used as a supplementary indicator for efficacy assessment.

## 4. Discussion

Recently, literature visualization has widely emerged as an excellent learning method that helps research scholars to systematically and vertically sort out the research base and development trend of a particular field and quickly capture the research hotspots as compared to the traditional literature review. This interdisciplinary approach has been accepted and applied by researchers in medical fields such as orthopedics and oncology.^[33–35]^ For example, Yuan Zhang et al conducted the first study of the literature related to RA-related interstitial lung disease, identified the most influential of 596 publications, and analyzed the current status and trends of research in this field.^[36]^ In recent years, the study of NLR by clinicians and specialists has been rapidly growing; however, there has not been a systematic combing of the literature and clinical validation related to NLR in RA to date. In order to fill this knowledge gap, we made a first attempt to take a literature visualization analysis perspective in order to gain a preliminary understanding of its knowledge structure and hot topics, especially the direction of its application in RA. The statistical and fitting analysis of 544 articles showed that the study of RA-NLR is attracting researchers’ interest and has a high prospect of development. The highly cited literature has addressed the key roles of neutrophils and lymphocytes in the pathogenesis and development of RA from multiple perspectives, respectively, demonstrating that the research on RA-NLR has a solid foundation. Our analysis of keywords further indicates that research in the field of RA-NLR is highly focused on terms such as inflammation, disease activity, therapy, diagnosis, and classification. Keyword clustering and timeline views revealed key clusters of “rheumatoid factor,” “autoantibodies,” and “regulatory effect,” suggesting that NLR may not only be related to clinical indicators such as RF and protein antibodies, but also potential studies focusing on efficacy assessment. In addition, keyword analysis identified key clusters such as “oxygen burst” and “extracellular traps.” Previous studies have shown that in the early stages of RA, large numbers of neutrophils pre-activated by immune complexes are recruited into synovial tissues and joints, and that infiltrating neutrophils exhibit an “activated” phenotype, including increased production of cytokines, chemokines and ROS, delayed apoptosis and formation of extracellular traps.^[37,38]^ The keyword outbreak emphasized that “disease activity,” “inflammation” and “classification” are still the key directions for future NLR research in RA. These results point to the direction of our next clinical retrospective studies.

Notably, some patients with RA do not yet show typical symptoms in the early stages, which increases the difficulty of diagnosis. By comparing the clinical presentation of patients with other rheumatic diseases and utilizing clinical RA patients to test NLR as an inflammatory marker, our study addresses an important and practical topic. We observed significantly higher levels of NLR, ESR, and CRP in RA patients than in non-RA patients, suggesting that a more severe immune imbalance and inflammatory state may exist in RA patients, similar to the study by Jin et al.^[39]^ After adjusting for confounders, our multifactorial logistic regression model showed that gender, age, NLR, ESR and CRP were independent predictors of RA. We also developed and validated a diagnostic nomogram combining NLR and other clinical risk factors to predict RA, which based on these predictors had an AUC value of 0.854, within an AUC of 0.736 for NLR, all of which showed good diagnostic value. This was further validated by the calibration curve and DCA. Recently, a meta-analysis by Mangoni et al^[40]^ confirmed that the AUC value of NLR for RA diagnosis was 0.76, with a combined sensitivity of 0.68 and specificity of 0.79, which is highly relevant to the results of this study. In addition, Chen et al^[41]^ suggested that NLR could be used as a novel biomarker for predicting and differentiating between patients with RA and RA-combined interstitial lung disease as well as healthy subjects. In contrast to these findings, our study proposes that for clinical rheumatology patients, NLR-based nomogram can help clinicians to identify RA high-risk groups early and give appropriate interventional therapy.

Assessing the severity of inflammatory activity in RA patients has long remained a challenging topic.^[42]^ A phenomenon exists here that in patients with low disease activity, indicators such as ESR, CRP, and RF are often at critical thresholds and can easily be overlooked. Earlier studies have also shown that some patients continue to experience progression of bone and joint degeneration even during clinical remission, which is attributed to persistent, underlying synovial inflammation.^[43]^ Therefore, there is an urgent need for an accurate and specific tool to assess RA disease activity and to treat the effects of therapy. NLR reflects the balance between neutrophils (inflammatory activators) and lymphocytes (inflammatory modulators). With the help of the cutoff value of NLR determined by the ROC curve, we observed that patients in the NLR > 2.258 group exhibited higher ESR, CRP, RF, and C3 levels and more severe VAS, PGA, PhGA, and CPRI-RA scores. Both association rule analysis and correlation analysis diaplayed that NLR > 2.258 was remarkably associated with ESR, CRP, RF, VAS, PGA, PhGA, PF, and BP, whereas gender was the most important factor affecting the baseline level of NLR. Fu et al^[44]^ also found that NLR was significantly increased in RA patients and was significantly and positively correlated with CRP, ESR, and disease activity score including the 28 Joint Score (DAS28). In contrast, Li et al^[45]^ reported that the NLR is of low value in differentiating between patients with inactive and active RA and may not be a useful independent diagnostic or complementary marker of disease activity in patients with RA, and the discrepancy in these findings may partly stem from differences in the study populations. Chandrashekara et al^[32]^ observed a significant increase in the NLR group in the areas of VAS, joint counts swelling (SJC-28), inflammatory parameters, and SF-36 indexes. These results all validate our literature visualization findings and are more consistent with previous reports.

Joint pain and activity limitation are the most intuitively felt by RA patients. The VAS score and CPRI-RA are the most routinely assessed tools for measuring informative symptoms and disease activity in RA patients. By constructing 4 stepwise-adjusted multivariate regression models, we found that NLR > 2.258 was a significant independent predictor of moderate-to-severe pain and higher disease activity. The stability and reliability of this predictive utility was further validated in subcomponent stratification analyses that incorporated each factor. In addition, a previous study found that NLR, as well as patient perception of pain, may help predict sustained remission with even better validity than CRP, ESR, and disease activity scores.^[46]^ Boulos et al^[47]^ identified higher NLR at baseline as an independent predictor of failure of triple therapy (methotrexate-sulfasalazine-hydroxychloroquine) in RA (OR = 2.65, *P* = .01, 95% CI = 1.23–5.72, AUG of 0.63), a predictive property that is unique to NLR and superior to traditional markers of disease activity. Lee et al^[48]^ demonstrated that a high baseline NLR (OR 5.57, *P* = .014) was significantly associated with a higher risk of non-response to anti-TNF-α medication at 12 weeks, as well as with an increased risk of anti-TNF-α discontinuation due to lack of efficacy (HR 2.12, *P* = .045). In contrast to these findings, our results emphasize that reduced NLR is highly associated with remission of ESR, CRP, RF, PF, BP, GH, VT, MH, PGA, CPRI-RA, SAS, and SDS. In conclusion, incorporating NLR into the clinical setting may provide a novel and practical approach to screening and intervening in individuals at risk for RA.

This study has several advantages. Firstly, it is based on massive literature and modern information technology to carry out exploration, and the research foundation and direction are reliable. Secondly, through clinical large-sample data investigation, we confirmed the unique value of NLR in RA diagnosis, disease assessment, and clinical remission, and we realized the mutual validation between the literature study and the clinical data study. However, our study has some limitations. It was a single-center, retrospective study that lacked longitudinal, dynamic testing, which may lead to biased results. Therefore, the predictive value of our model in long-term prognosis may be further confirmed in the future by a prospective, multicenter cohort study.

## 5. Conclusion

In conclusion, based on the dual perspective of literature studies and clinical retrospective data, we found that RA-NLR-related studies focused on inflammation, disease activity, and diagnostic value, and specifically, NLR was significantly associated with moderate-to-severe pain, high disease activity, and remission prognosis in patients with RA. This study supports the role of NLR in identifying, predicting, and intervening in individuals at high risk for RA.

## Acknowledgments

The authors take thankful pleasure in acknowledging the unsparing assistance of all participants.

## Author contributions

**Conceptualization:** Jian Liu.

**Data curation:** Yuedi Hu.

**Formal analysis:** Yang Li.

**Funding acquisition:** Jian Liu.

**Investigation:** Yuedi Hu, Chengzhi Cong, Yiming Chen.

**Methodology:** Yang Li.

**Project administration:** Jian Liu.

**Validation:** Yanyan Fang.

**Writing – original draft:** Yang Li.

**Writing – review & editing:** Yang Li, Jian Liu.

## Supplementary Material



## References

[1] Yu B Chen Y Chen E. LncRNA RNA XIST binding to GATA1 contributes to rheumatoid arthritis through its effects on proliferation of synovial fibroblasts and angiogenesis via regulation of CCN6. Mol Immunol. 2023;153:200–11. 36542956 10.1016/j.molimm.2022.12.004

[2] van der Woude D van der Helm-van Mil AHM. Update on the epidemiology, risk factors, and disease outcomes of rheumatoid arthritis. Best Pract Res Clin Rheumatol. 2018;32:174–87. 30527425 10.1016/j.berh.2018.10.005

[3] Cush JJ. Rheumatoid arthritis: early diagnosis and treatment. Rheum Dis Clin North Am. 2022;48:537–47. 35400377 10.1016/j.rdc.2022.02.010

[4] Wang J Xue Y Zhou L. Comparison of immune cells and diagnostic markers between spondyloarthritis and rheumatoid arthritis by bioinformatics analysis. J Transl Med. 2022;20:196. 35509008 10.1186/s12967-022-03390-y PMC9066892

[5] He Y Tang J Wu B Yang B Ou Q Lin J. Correlation between albumin to fibrinogen ratio, C-reactive protein to albumin ratio and Th17 cells in patients with rheumatoid arthritis. Clin Chim Acta. 2020;500:149–54. 31672633 10.1016/j.cca.2019.10.009

[6] Orr CK Najm A Young F. The utility and limitations of CRP, ESR and DAS28-CRP in appraising disease activity in rheumatoid arthritis. Front Med (Lausanne). 2018;5:185. 30123796 10.3389/fmed.2018.00185 PMC6085449

[7] O’Neil LJ Kaplan MJ. Neutrophils in rheumatoid arthritis: breaking immune tolerance and fueling disease. Trends Mol Med. 2019;25:215–27. 30709614 10.1016/j.molmed.2018.12.008

[8] Alivernini S Tolusso B Fedele AL Di Mario C Ferraccioli G Gremese E. The B side of rheumatoid arthritis pathogenesis. Pharmacol Res. 2019;149:104465. 31574298 10.1016/j.phrs.2019.104465

[9] Lübbers J van Beers-Tas MH Vosslamber S. Changes in peripheral blood lymphocyte subsets during arthritis development in arthralgia patients. Arthritis Res Ther. 2016;18:205. 27629388 10.1186/s13075-016-1102-2 PMC5024500

[10] Erre GL Paliogiannis P Castagna F. Meta-analysis of neutrophil-to-lymphocyte and platelet-to-lymphocyte ratio in rheumatoid arthritis. Eur J Clin Invest. 2019;49:e13037. 30316204 10.1111/eci.13037

[11] Teitsma XM Jacobs JWG Welsing PMJ. Patient-reported outcomes in newly diagnosed early rheumatoid arthritis patients treated to target with a tocilizumab- or methotrexate-based strategy. Rheumatology (Oxford). 2017;56:2179–89. 29029185 10.1093/rheumatology/kex319

[12] Bacci ED DeLozier AM Lin C-Y. Psychometric properties of morning joint stiffness duration and severity measures in patients with moderately to severely active rheumatoid arthritis. Health Qual Life Outcomes. 2017;15:239. 29212515 10.1186/s12955-017-0813-7 PMC5719576

[13] Wen J Liu J Wang B. Prediction of self-perception of patient in rheumatoid arthritis with the key RNAs expression profiles. Front Med (Lausanne). 2020;7:567. 33072778 10.3389/fmed.2020.00567 PMC7530568

[14] Hu Y Liu J Xin L. Huangqin Qingre Chubi capsule is associated with reduced risk of readmission in patients with rheumatoid arthritis: a real-world retrospective cohort study. Int J Gen Med. 2023;16:4819–34. 37908759 10.2147/IJGM.S431124 PMC10615257

[15] Oude Voshaar MAH Das Gupta Z Bijlsma JWJ. International consortium for health outcome measurement set of outcomes that matter to people living with inflammatory arthritis: consensus from an International Working Group. Arthritis care & research. 2019;71:1556–65. 30358135 10.1002/acr.23799 PMC6900179

[16] Wen P Luo P Zhang B Zhang Y. Mapping knowledge structure and global research trends in gout: a bibliometric analysis from 2001 to 2021. Front Public Health. 2022;10:924676. 35844867 10.3389/fpubh.2022.924676 PMC9277182

[17] Xu Y Zhang Z He J Chen Z. Immune effects of macrophages in rheumatoid arthritis: a bibliometric analysis from 2000 to 2021. Front Immunol. 2022;13:903771. 36172378 10.3389/fimmu.2022.903771 PMC9510364

[18] Chen T Zhu J Zhao Y. The global state of research in pain management of osteoarthritis (2000–2019): a 20-year visualized analysis. Medicine (Baltimore). 2021;100:e23944. 33466135 10.1097/MD.0000000000023944 PMC7808549

[19] van Eck NJ Waltman L. Software survey: VOSviewer, a computer program for bibliometric mapping. Scientometrics. 2010;84:523–38. 20585380 10.1007/s11192-009-0146-3 PMC2883932

[20] Synnestvedt MB Chen C Holmes JH. CiteSpace II: visualization and knowledge discovery in bibliographic databases. AMIA Annu Symp Proc. 2005;2005:724–8. 16779135 PMC1560567

[21] Pedersen E Pinsker E Younger ASE. Outcome of total ankle arthroplasty in patients with rheumatoid arthritis and noninflammatory arthritis: a multicenter cohort study comparing clinical outcome and safety. J Bone Joint Surg Am. 2014;96:1768–75. 25378503 10.2106/JBJS.M.01164

[22] Matcham F Norton S Steer S Hotopf M. Usefulness of the SF-36 Health Survey in screening for depressive and anxiety disorders in rheumatoid arthritis. BMC Musculoskelet Disord. 2016;17:224. 27215696 10.1186/s12891-016-1083-y PMC4878044

[23] Zhong LL Wang R Lam WC. The combination of Chinese and Western Medicine in the management of rheumatoid arthritis: a real-world cohort study across China. Front Pharmacol. 2022;13:933519. 36278204 10.3389/fphar.2022.933519 PMC9582451

[24] Boeren AMP Verstappen M Looijen AEM de Jong PHP van der Helm-van Mil AHM. Patients with rheumatoid arthritis presenting with mono- or oligo-arthritis and high VAS-ratings remain the most fatigued during 5 years of follow-up. Rheumatology (Oxford). 2024;63:1574–81. 37632771 10.1093/rheumatology/kead429 PMC11147540

[25] Han M Liu H Tang X. Expert consensus of clinical outcomes reported by Chinese rheumatoid arthritis patients. J Tradit Chin Med. 2018;59:897–900.

[26] Liu L Zhu F Xin Y. Real-world effects of Yishen Tongbi decoction for rheumatoid arthritis: protocol for a prospective, observational, multicenter cohort study with validation against double-blind, randomized, controlled trial. Front Pharmacol. 2024;15:1320578. 38410132 10.3389/fphar.2024.1320578 PMC10895057

[27] Zhang C Wu X Yuan Y. Effect of solution-focused approach on anxiety and depression in patients with rheumatoid arthritis: a quasi-experimental study. Front Psychol. 2022;13:939586. 36582330 10.3389/fpsyg.2022.939586 PMC9792673

[28] Wright HL Moots RJ Edwards SW. The multifactorial role of neutrophils in rheumatoid arthritis. Nat Rev Rheumatol. 2014;10:593–601. 24914698 10.1038/nrrheum.2014.80

[29] Carmona-Rivera C Carlucci PM Moore E. Synovial fibroblast-neutrophil interactions promote pathogenic adaptive immunity in rheumatoid arthritis. Sci Immunol. 2017;2:aag3358. 10.1126/sciimmunol.aag3358 PMC5479641 28649674

[30] Lundy SK Sarkar S Tesmer LA Fox DA. Cells of the synovium in rheumatoid arthritis. T lymphocytes. Arthritis Res Ther. 2007;9:202. 17306038 10.1186/ar2107 PMC1860060

[31] Uslu AU Küçük A Şahin A. Two new inflammatory markers associated with Disease Activity Score-28 in patients with rheumatoid arthritis: neutrophil-lymphocyte ratio and platelet-lymphocyte ratio. Int J Rheum Dis. 2015;18:731–5. 25900081 10.1111/1756-185X.12582

[32] Chandrashekara S Mukhtar Ahmad M Renuka P Anupama KR Renuka K. Characterization of neutrophil-to-lymphocyte ratio as a measure of inflammation in rheumatoid arthritis. Int J Rheum Dis. 2017;20:1457–67. 28952205 10.1111/1756-185X.13157

[33] Zhang Y Wang Y Chen J. The top 100 cited articles in osteonecrosis of the femoral head: a bibliometric analysis. Biomed Res Int. 2021;2021:1433684. 34462719 10.1155/2021/1433684 PMC8403054

[34] Xu Q Zhou Y Zhang H Li H Qin H Wang H. Bibliometric analysis of hotspots and frontiers of immunotherapy in pancreatic cancer. Healthcare (Basel). 2023;11:304. 36766879 10.3390/healthcare11030304 PMC9914338

[35] Pappen E Morschbacher AP Granada CE Contini V Henriques JAP. Evolution of the scientific literature on esophageal cancer from 1945 to 2020: a bibliometric analysis. An Acad Bras Cienc. 2023;95:e20220716. 36790272 10.1590/0001-3765202320220716

[36] Zhang Y Zhao T Wu T. Bibliometric analysis of the scientific literature on rheumatoid arthritis-associated interstitial lung disease. Biomed Res Int. 2021;2021:7899929. 34966821 10.1155/2021/7899929 PMC8712181

[37] Wright HL Lyon M Chapman EA Moots RJ Edwards SW. Rheumatoid arthritis synovial fluid neutrophils drive inflammation through production of chemokines, reactive oxygen species, and neutrophil extracellular traps. Front Immunol. 2020;11:584116. 33469455 10.3389/fimmu.2020.584116 PMC7813679

[38] Pan W Xin Q Xu J. IgD enhances the release of neutrophil extracellular traps (NETs) via FcδR in rheumatoid arthritis patients. Int Immunopharmacol. 2023;114:109484. 36450207 10.1016/j.intimp.2022.109484

[39] Jin Z Cai G Zhang P. The value of the neutrophil-to-lymphocyte ratio and platelet-to-lymphocyte ratio as complementary diagnostic tools in the diagnosis of rheumatoid arthritis: a multicenter retrospective study. J Clin Lab Anal. 2021;35:e23569. 32951253 10.1002/jcla.23569 PMC7843258

[40] Mangoni AA Zinellu A. Diagnostic accuracy of the neutrophil-to-lymphocyte ratio and the platelet-to-lymphocyte ratio in rheumatoid arthritis: a systematic review and meta-analysis. Clin Exp Med. 2024;24:207. 39230596 10.1007/s10238-024-01478-x PMC11374877

[41] Chen Q Chen DY Xu XZ Liu Y-Y Yin T-T Li D. Platelet/lymphocyte, lymphocyte/monocyte, and neutrophil/lymphocyte ratios as biomarkers in patients with rheumatoid arthritis and rheumatoid arthritis-associated interstitial lung disease. Med Sci Monit. 2019;25:6474–81. 31462627 10.12659/MSM.916583 PMC6733153

[42] Coutant F Miossec P. Evolving concepts of the pathogenesis of rheumatoid arthritis with focus on the early and late stages. Curr Opin Rheumatol. 2020;32:57–63. 31644463 10.1097/BOR.0000000000000664

[43] Yu X Li Z Ren M Xi J Wu J Ji Y. Superb microvascular imaging (SMI) for evaluating hand joint lesions in patients with rheumatoid arthritis in clinical remission. Rheumatol Int. 2018;38:1885–90. 30062435 10.1007/s00296-018-4112-3 PMC6132695

[44] Fu H Qin B Hu Z. Neutrophil- and platelet-to-lymphocyte ratios are correlated with disease activity in rheumatoid arthritis. Clin Lab. 2015;61:269–73. 25974992 10.7754/clin.lab.2014.140927

[45] Lijuan W Yuting Z Chaoyang L Ju Y. Neutrophil-lymphocyte, platelet-lymphocyte and lymphocyte-monocyte ratios may not be useful markers to assess disease activity in rheumatoid arthritis: a STROBE-compliant article. Medicine (Baltimore). 2021;100:e27631. 34766563 10.1097/MD.0000000000027631 PMC8589242

[46] Chandrashekara S Rajendran A Bai Jaganath A Krishnamurthy R. Neutrophil-lymphocyte ratio, pain perception, and disease activity score may serve as important predictive markers for sustained remission in rheumatoid arthritis. Reumatismo. 2015;67:109–15. 26876190 10.4081/reumatismo.2015.838

[47] Boulos D Proudman SM Metcalf RG McWilliams L Hall C Wicks IP. The neutrophil-lymphocyte ratio in early rheumatoid arthritis and its ability to predict subsequent failure of triple therapy. Semin Arthritis Rheum. 2019;49:373–6. 31248587 10.1016/j.semarthrit.2019.05.008

[48] Lee HN Kim YK Kim GT. Neutrophil-to-lymphocyte and platelet-to-lymphocyte ratio as predictors of 12-week treatment response and drug persistence of anti-tumor necrosis factor-α agents in patients with rheumatoid arthritis: a retrospective chart review analysis. Rheumatol Int. 2019;39:859–68. 30874873 10.1007/s00296-019-04276-x

