# Supplementary material for: The neutrophil-to-lymphocyte ratio in rheumatoid arthritis: The dual perspectives from literature and clinic
Source: Medicine (Baltimore). 2025 Sep 19;104(38):e44554. doi: 10.1097/MD.0000000000044554 (PMC12459527; doi:10.1097/MD.0000000000044554)
Supplement: Supplementary file 1 [file medi-104-e44554-s001.docx]

Table S1 Top 10 references with the most co-citations

| Rank | Co-cited reference | Citations | Total link strength |
| --- | --- | --- | --- |
| 1 | arnett fc, 1988, arthritis rheum, v31, p315, doi 10.1002/art.1780310302 | 181 | 764 |
| 2 | wright hl, 2014, nat rev rheumatol, v10, p593, doi 10.1038/nrrheum.2014.80 | 49 | 715 |
| 3 | khandpur r, 2013, sci transl med, v5, doi 10.1126/scitranslmed.3005580 | 48 | 910 |
| 4 | prevoo mll, 1995, arthritis rheum, v38, p44, doi 10.1002/art.1780380107 | 43 | 221 |
| 5 | aletaha d, 2010, arthritis rheum-us, v62, p2569, doi 10.1002/art.27584 | 32 | 231 |
| 6 | mcinnes ib, 2011, new engl j med, v365, p2205, doi 10.1056/nejmra1004965 | 31 | 335 |
| 7 | aletaha d, 2010, ann rheum dis, v69, p1580, doi 10.1136/ard.2010.138461 10.1002/art.27584 | 29 | 165 |
| 8 | wipke bt, 2001, j immunol, v167, p1601, doi 10.4049/jimmunol.167.3.1601 | 27 | 398 |
| 9 | brinkmann v, 2004, science, v303, p1532, doi 10.1126/science.1092385 | 26 | 525 |
| 10 | wright hl, 2010, rheumatology, v49, p1618, doi 10.1093/rheumatology/keq045 | 24 | 361 |

Table S2 The top 20 most-cited publications on RA-NLR

| Rank | Title | First Author | Journal | JIF (2023) | Publication year | Total citations | Type |
| --- | --- | --- | --- | --- | --- | --- | --- |
| 1 | Essential role of neutrophils in the initiation and progression of a murine model of rheumatoid arthritis | Wipke, BT | The Journal of Immunology | 4.4 | 2001 | 487 | Article |
| 2 | Sustained improvement in rheumatoid arthritis following a protocol designed to deplete B lymphocytes | Edwards, JCW | Rheumatology | 5.5 | 2001 | 412 | Article |
| 3 | Serologic changes following B lymphocyte depletion therapy for rheumatoid arthritis | Cambridge, G | Arthritis & Rheumatology | 13.3 | 2003 | 376 | Article |
| 4 | The multifactorial role of neutrophils in rheumatoid arthritis | Wright, HL | Nature Reviews Rheumatology | 33.7 | 2014 | 373 | Review |
| 5 | In vivo production of interleukin-10 by non–t cells in rheumatoid arthritis, sjöugren's syndrome, and systemic lupus erythematosus | Llorente, L | Arthritis & Rheumatology | 13.3 | 1994 | 319 | Article |
| 6 | Seeing the wood for the trees: the forgotten role of neutrophils in rheumatoid arthritis | Edwards, SW | Immunology Today | 12.856 | 1997 | 288 | Article |
| 7 | Clinical outcome in 22 patients with rheumatoid arthritis treated with B lymphocyte depletion | Leandro, MJ | Annals of the Rheumatic Diseases | 27.4 | 2002 | 270 | Article |
| 8 | Shift toward T lymphocytes with a T helper 1 cytokine-secretion profile in the joints of patients with rheumatoid arthritis | Dolhain, RJEM | Arthritis & Rheumatology | 13.3 | 1996 | 259 | Article |
| 9 | Circulating levels of B lymphocyte stimulator in patients with rheumatoid arthritis following rituximab treatment - Relationships with B cell depletion, circulating antibodies, and clinical relapse | Cambridge, G | Arthritis & Rheumatology | 13.3 | 2006 | 220 | Article |
| 10 | IL-33 induces neutrophil migration in rheumatoid arthritis and is a target of anti-TNF therapy | Verri, WA | Annals of the Rheumatic Diseases | 27.4 | 2010 | 210 | Article |
| 11 | CD4+CD28-T lymphocytes contribute to early atherosclerotic damage in rheumatoid arthritis patients | Gerli, R | Circulation | 37.8 | 2004 | 205 | Article |
| 12 | Evidence that anti-tumor necrosis factor therapy with both etanercept and infliximab induces apoptosis in macrophages, but not lymphocytes, in rheumatoid arthritis joints | Catrina, AI | Arthritis & Rheumatology | 13.3 | 2005 | 201 | Article |
| 13 | A large subset of neutrophils expressing membrane proteinase 3 is a risk factor for vasculitis and rheumatoid arthritis | Witko-Sarsat, V | Journal of the American Society of Nephrology | 13.6 | 1999 | 190 | Article |
| 14 | Synovial fibroblast-neutrophil interactions promote pathogenic adaptive immunity in rheumatoid arthritis | Carmona-Rivera, C | Science Immunology | 24.8 | 2017 | 181 | Article |
| 15 | MiR-223 is overexpressed in T-lymphocytes of patients affected by rheumatoid arthritis | Fulci, V | Human Immunology | 2.7 | 2010 | 174 | Article |
| 16 | Antibodies from patients with rheumatoid arthritis target citrullinated histone 4 contained in neutrophils extracellular traps | Pratesi, F | Annals of the Rheumatic Diseases | 27.4 | 2014 | 173 | Article |
| 17 | Cells of the synovium in rheumatoid arthritis - T lymphocytes | Lundy, SK | Arthritis Research & Therapy | 4.9 | 2007 | 171 | Review |
| 18 | Neutrophils in rheumatoid arthritis: More than simple final effectors | Cascao, R | Autoimmunity Reviews | 13.6 | 2010 | 168 | Review |
| 19 | Release of Active Peptidyl Arginine Deiminases by Neutrophils Can Explain Production of Extracellular Citrullinated Autoantigens in Rheumatoid Arthritis Synovial Fluid | Spengler, J | Arthritis & Rheumatology | 13.3 | 2015 | 162 | Article |
| 20 | Two new inflammatory markers associated with Disease Activity Score-28 in patients with rheumatoid arthritis: neutrophil-lymphocyte ratio and platelet-lymphocyte ratio | Uslu, AU | International Journal of Rheumatic Diseases | 2.5 | 2015 | 147 | Article |

Table S3 Correlation between NLR, laboratory indicators and SPP scores

| Variables | | NLR | |
| --- | --- | --- | --- |
|  |  | r | *P* value |
| Laboratory index | ESR | 0.200 | <0.001 |
|  | CRP | 0.393 | <0.001 |
|  | RF | 0.114 | 0.001 |
|  | CCP | 0.019 | 0.584 |
|  | IgA | 0.048 | 0.168 |
|  | IgG | -0.121 | <0.001 |
|  | IgM | -0.023 | 0.509 |
|  | C3 | 0.156 | <0.001 |
|  | C4 | 0.050 | 0.151 |
| Self-perception of patients | PF | -0.069 | 0.046 |
|  | RP | 0.040 | 0.254 |
|  | BP | -0.069 | 0.046 |
|  | GH | -0.059 | 0.091 |
|  | VT | -0.013 | 0.700 |
|  | SF | -0.036 | 0.300 |
|  | RE | 0.021 | 0.545 |
|  | MH | -0.020 | 0.562 |
|  | HT | 0.037 | 0.290 |
|  | VAS | 0.110 | 0.001 |
|  | PGA | 0.090 | 0.009 |
|  | PhGA | 0.099 | 0.004 |
|  | CPRI-RA | 0.092 | 0.008 |
|  | SAS | 0.032 | 0.350 |
|  | SDS | 0.059 | 0.087 |

Table S4 Association rule analysis of reduction of NLR and reduction of immune inflammation index and recovery of SPP score

| Items (Antecedent ⇒ Consequent) | Support (%) | Confidence (%) | Lift |
| --- | --- | --- | --- |
| NLR↓ ⇒ ESR↓ | 45.75 | 76.70 | 1.251 |
| NLR↓ ⇒ CRP↓ | 45.75 | 67.54 | 1.187 |
| NLR↓ ⇒ RF↓ | 45.75 | 65.71 | 1.150 |
| NLR↓ ⇒ PF↑ | 45.75 | 82.72 | 1.020 |
| NLR↓ ⇒ BP↑ | 45.75 | 73.82 | 1.019 |
| NLR↓ ⇒ GH↑ | 45.75 | 73.04 | 1.034 |
| NLR↓ ⇒ VT↑ | 45.75 | 77.75 | 1.030 |
| NLR↓ ⇒ MH↑ | 45.75 | 76.70 | 1.030 |
| NLR↓ ⇒ PGA↓ | 45.75 | 98.43 | 1.006 |
| NLR↓ ⇒ CPRI-RA↓ | 45.75 | 91.36 | 1.017 |
| NLR↓ ⇒ SAS↓ | 45.75 | 90.58 | 1.010 |
| NLR↓ ⇒ SDS↓ | 45.75 | 89.27 | 1.022 |

Notes: ↑ indicates a downward adjustment; ↓ indicates a downward adjustment.

Table S5 Association analysis between the NLR reduction and reduced laboratory indicators and recovery of SPP score

| Variables | | NLR | |
| --- | --- | --- | --- |
|  |  | r | *P* value |
| Laboratory index | ESR | 0.177 | <0.001 |
|  | CRP | 0.187 | <0.001 |
|  | RF | 0.119 | 0.001 |
|  | CCP | 0.056 | 0.109 |
|  | IgA | 0.112 | 0.001 |
|  | IgG | 0.086 | 0.013 |
|  | IgM | 0.093 | 0.007 |
|  | C3 | 0.141 | <0.001 |
|  | C4 | 0.077 | 0.026 |
| Self-perception of patients | PF | 0.012 | 0.739 |
|  | RP | 0.013 | 0.717 |
|  | BP | -0.019 | 0.596 |
|  | GH | 0.064 | 0.064 |
|  | VT | 0.027 | 0.432 |
|  | SF | -0.007 | 0.845 |
|  | RE | 0.022 | 0.521 |
|  | MH | 0.018 | 0.604 |
|  | HT | -0.004 | 0.904 |
|  | VAS | 0.002 | 0.952 |
|  | PGA | -0.007 | 0.842 |
|  | PhGA | -0.022 | 0.087 |
|  | CPRI-RA | 0.059 | 0.202 |
|  | SAS | 0.044 | 0.189 |
|  | SDS | 0.046 | 0.521 |
